# Rapid access to the core of malayamycin A by intramolecular dipolar cycloaddition

**DOI:** 10.3762/bjoc.21.196

**Published:** 2025-11-17

**Authors:** Yilin Liu, Yuchen Yang, Chen Yang, Sha-Hua Huang, Jian Jin, Ran Hong

**Affiliations:** 1 Faculty of Chemical Engineering and Energy Technology, Shanghai Institute of Technology, 100 Haiquan Road, Shanghai 201418, P.R. China; 2 State Key Laboratory of Chemical Biology, Shanghai Institute of Organic Chemistry, Chinese Academy of Sciences, 345 Lingling Road, Shanghai 200032, P.R. China

**Keywords:** dipolar cycloaddition, elimination, fungicide, nucleoside, oxazoline

## Abstract

We have streamlined a dipolar cycloaddition approach to assemble the core of malayamycin A and other related uracil nucleosides possessing the common bicyclic perhydrofuropyran framework. The latent functionality strategy employing oxazoline to unmask the 1,2-hydroxyamine moiety proves feasible, eliminating the need for alkene functionalization required in previous endeavours. This current strategy provides a reliable platform for accessing diverse uracil nucleosides and their derivatives, facilitating the development of potent fungicides.

## Introduction

Modern agriculture relies on various effective fungicides to combat crop diseases for achieving significant gains [1]. However, the long-term and widespread use of chemicals and biological agents has led to a rapid emergence of resistance, which in turn diminishes the national-wide and even global-wide food security. Moreover, toxins produced by fungi in diseased crops have serious impacts on animal and human health [2]. There remains high demand to develop new antifungal compounds as alternatives to existing fungicides as well as enhancing the control spectrum and persistence [3].

**Figure 1:**
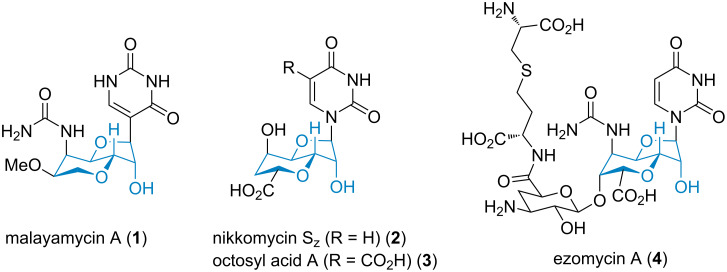
Selected natural uracil-containing nucleosides (the key perhydrofuropyran core highlighted in blue).

A group of scientists at Syngenta reported a bicyclic perhydrofuropyran C-nucleoside malayamycin A (**1**) from the soil bacterium *Streptomyces malaysiensis* [4] (Figure 1). This novel compound was found to inhibit the sporulation of *Stagonospora nodorum* (Berk) Castell and Germano that were identified as the culprit of wheat glume blotch disease [5]. Furthermore, malayamycin functions as a broad-spectrum fungicide with an unusually higher potency in planta than in vitro, suggesting that its mode of action may not be consistent with those of known fungicide classes. This encouraging characteristic indicates its potential to overcome resistance to other fungicides [6–8].

Structurally, malayamycin A belongs to a class of modified nucleosides that mimic UDP (uridine 5′-diphosphate)-linked metabolites and exhibit intriguing bioactivities (**2**–**4**, Figure 1). These antifungal nucleoside agents have been received great attention from the scientific community and several elegant syntheses have been disclosed [9–15]. Specifically, Hanessian and co-workers reported the first total synthesis and structural determination of malaymycin A (**1**) as well as the subsequent design of structural analogues for biological evaluation [16–20]. Preliminary structure–activity relationship (SAR) studies revealed that fungicidal activity is highly dependent on the nature and stereochemistry of substituents, as well as the heterocyclic anomeric unit. Uncertainty regarding the mode of action, along with inadequate synthetic approaches toward a lead compound, remains elusive.

**Scheme 1:**
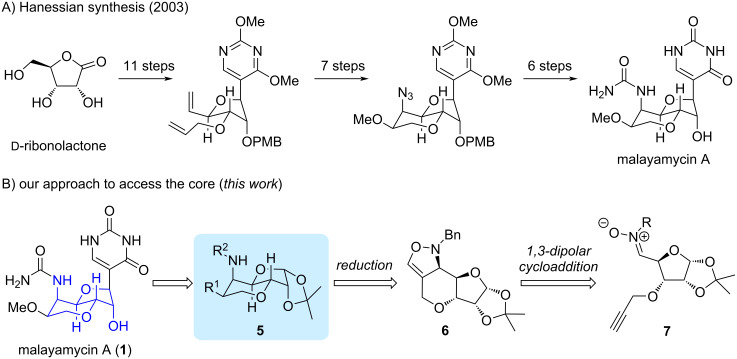
Synthetic strategies toward malayamycin A. (A) Previous synthetic route. (B) Our strategy toward the core skeleton.

The well-established synthetic route reported by Hanessian and co-workers began with ᴅ-ribonolactone which bears three contiguous stereogenic centers (Scheme 1A). The pyran ring was constructed by a RCM reaction [16]. Subsequent functionalization of the alkene to install the 1,2-*cis*-hydroxy amine required 6 steps from the sterically more demanding side. In continuing our recent interest in accessing unusual monosaccharides and applying dipolar cycloaddition to construct various bioactive compounds [21–27], we intended to develop a practical strategy to access the perhydrofuropyran core of malayamycin A and other uracil nucleosides to enable future rapid derivatization (Scheme 1B). The bicyclic intermediate **5** will be converted into the final target after installation of the urea and uracil motifs. Accordingly, a nitrone-based latent functionality approach [28] would be tunable from fully substituted tetrahydrofuran-derived nitrone **7**. The *cis*-1,2-hydroxy amine could be derived from oxazoline **6** through cleavage of the N–O bond, oxidation and Baeyer–Villiger oxidation. The starting functional groups (including alkyne and nitrone) for the proposed oxazoline were established in literature precedents [29–31]. Moreover, the readily available intermediate **8** [32] bearing three defined stereogenic centers is secured from the commercial source.

## Results and Discussion

**Scheme 2:**
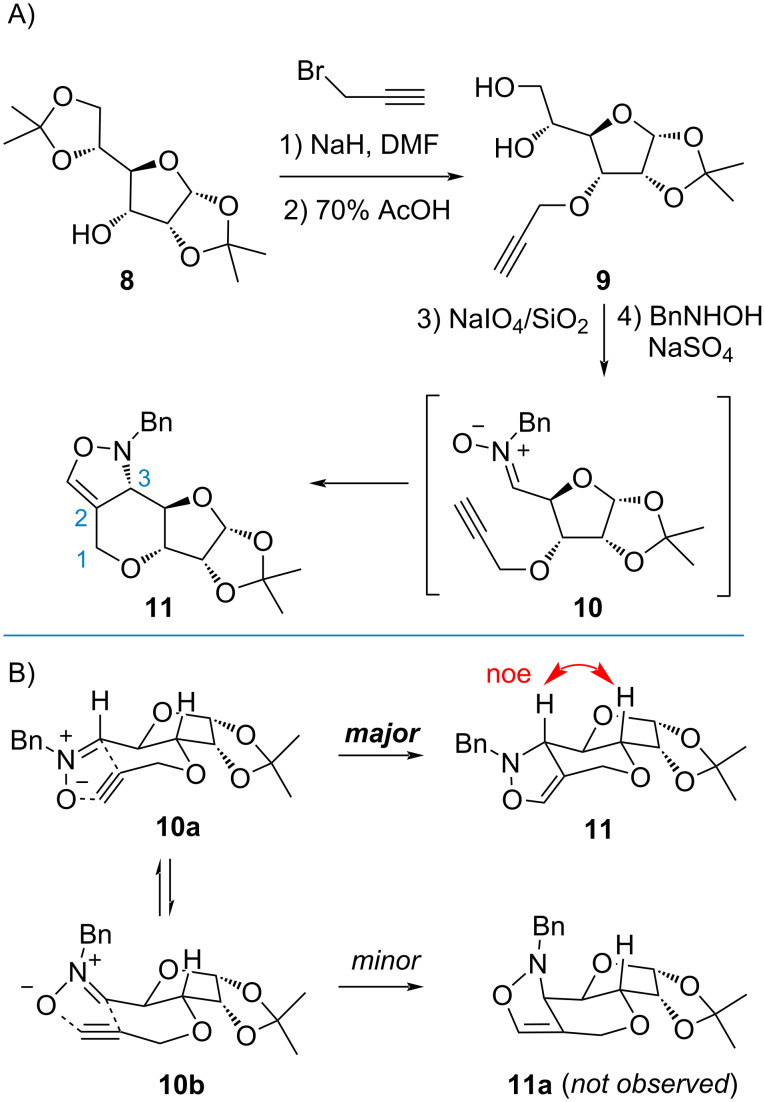
Rational for intramolecular dipolar cycloaddition.

**Scheme 3:**
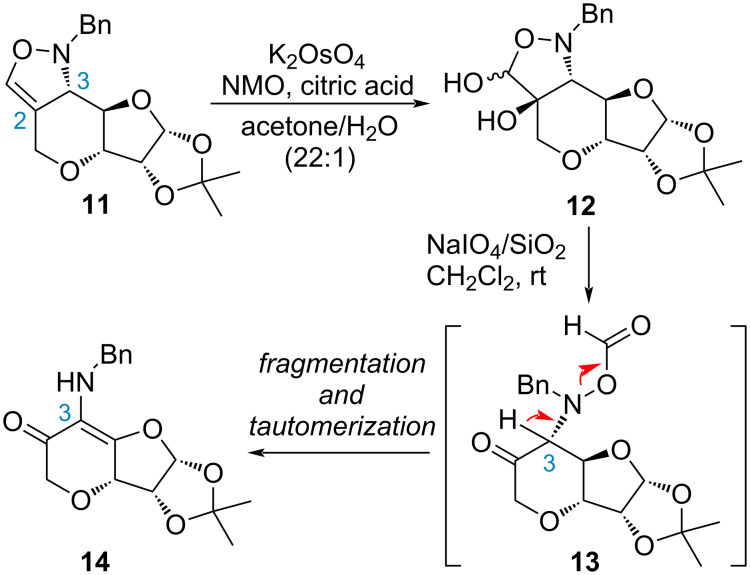
Proposed pathway for the enone formation.

Based on the known protocol [33], diacetone-ᴅ-allofuranose **8** was first introduced with a propargyl group (Scheme 2A). Upon treatment of AcOH to afford diol **9**, oxidative cleavage with Shing’s protocol (NaIO_4_ on silica gel) [34] proceeded smoothly to deliver the aldehyde which was immediately subjected to the condensation reaction with benzylhydroxylamine. The corresponding nitrone **10** then underwent an intramolecular cycloaddition. Adduct **11** was isolated as the major product in 42% yield for 2 steps. Comprehensive NMR analysis revealed the undesired stereochemistry at C3 due to a possible chair-like transition state like **10a** (Scheme 2B). This phenomenon is consistent with the observations from previous syntheses [31,35–36]. We anticipated that late-stage epimerization might invert the configuration once the acyl group is revealed at the C2 position. Therefore, dihydroxylation [37] readily converted alkene **11** to diol **12** as a mixture of inseparable isomers. Without purification, oxidative cleavage with NaIO_4_ resulted in a compound with strong UV absorption, which was eventually identified as enone **14** (Scheme 3). It is assumed that the formyloxy group on the N atom in the unstable intermediate **13** serves as electron-withdrawing group to facilitate fragmentation when removal of the acidic proton at C2 was initiated.

**Scheme 4:**
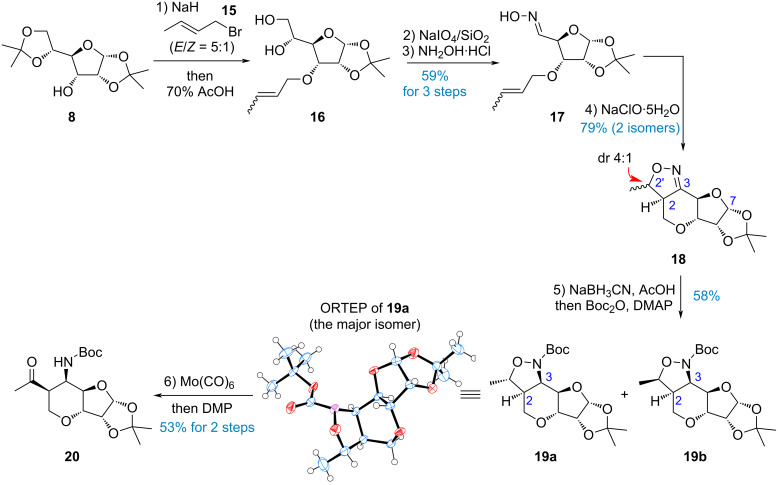
Modified route to access the core of malayamycins.

Although the reduction of enone **14** could provide the requisite stereoisomer, the rigid conformation of such bicyclic [4.3.0]-ring necessitates tedious optimization to properly install three continuous tertiary centers (C2, C3, and C4). To circumvent the influence of the electron-withdrawing group on the nitrogen atom, we anticipated that cleaving the N–O bond prior to breaking the C2–C2’ bond would be feasible. However, several conditions to cleave the N–O bond in **11** or **12** had not yielded any successful outcome. Therefore, we turned to adjust the synthetic sequence to switch the oxidation states at C2 and C3 (Scheme 4). It should be noted that compound **16** [38] was obtained as a stereoisomeric mixture of olefin, resulting from the use of crotyl bromide as a mixture of geometric isomers. After installation of the crotyl group, hydrolysis of the acetonide group and oxidative cleavage of diol **16**, oxime **17** was prepared through the condensation of the aldehyde with hydroxylamine in overall 59% yield. Upon oxidation with NaClO·5H_2_O, the in situ-generated nitrile *N*-oxide immediately underwent intramolecular dipolar cycloaddition to deliver the cycloadducts **18** in good yield as a mixture of two inseparable diastereoisomers. This telescoped step was readily performed on a gram scale without interrupted purification of the nitrile oxide.

At this stage, it is not clear that the diastereomeric ratio (dr 4:1) may share with the same configuration at the C2 or C2’ position. The mixture of **18** was subjected to reduction of the imine motif by NaBH_3_CN in AcOH–MeOH and immediately protected with the Boc group. The isolated yield of **19** was moderate because the anomeric carbon at C7 is associated with an acid-sensitive acetonide group. The structural determination of the major product **19a** was accomplished by comprehensive NMR spectroscopy and further unambiguously confirmed by X-ray analysis (see Supporting Information File 1 for details). The following reductive cleavage of the N–O bond was carried out by Mo(CO)_6_ in refluxing CH_3_CN [39]. Subsequent oxidation of the resulting secondary alcohol with Dess–Martin periodinane (DMP) [40] afforded methyl ketone **20** in 53% yield for 2 steps. Moreover, the minor isomer **19b** also underwent the above two-step sequence, yielding a product identical to **20** (the synthetic route is not shown). This indicates that the pair of diastereomers essentially differs only at the C2’ position arised from the isomeric mixture of crotyl bromide **15**. It is also worth noting that both stereoisomers derived from the INOC cycloaddition can be converted to a single stereoisomer of **20**.

**Scheme 5:**
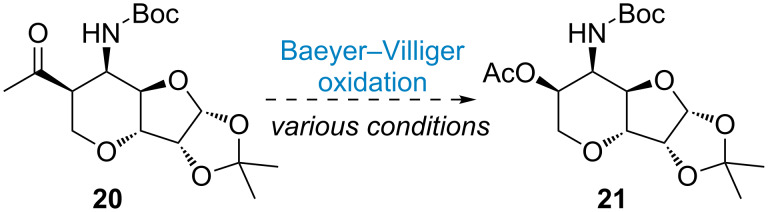
Attempting the Baeyer–Villiger reaction.

With all required stereogenic centers embedded in the 6-5 *trans*-fused bicyclic skeleton, the remaining problem is converting C to O to install the secondary alcohol at C2 with retention of the β-configuration. The programmed Baeyer–Villiger (BV) oxidation would be a feasible transformation to furnish all necessary functional groups for the completion of the core skeleton in malayamycins. To our surprise, it turns out very challenging for the BV oxidation. Several oxidants were examined and the desired acetate **21** remains inaccessible (Scheme 5) [41]. We assumed the steric hinderance of the Boc protecting group might have a great impact to the reactivity of ketone. Investigation along this line is currently on the way.

## Conclusion

In summary, we have streamlined the rapid construction of the core perhydrofuropyran skeleton of malayamycin A and other related uracil nucleosides via a dipolar cycloaddition. The latent functionality strategy employing oxazoline to reveal the *cis*-1,2-hydroxyamine moiety proves to be feasible, circumventing the lengthy route for alkene functionalization required in previous syntheses. Although the target-oriented synthesis toward malayamycins remains to be complished and several steps need to be improved, the current strategy provides a reliable platform to access various uracil nucleosides and derivatives for developing potent fungicides.

## Supporting Information

File 1 Experimental procedures and compound characterization data.

## Data Availability

Data generated and analyzed during this study is available from the corresponding author upon reasonable request.

## References

[1] Steffens J J, Pell E J, Tien M (1996). Curr Opin Biotechnol.

[2] Yin Y, Miao J, Shao W, Liu X, Zhao Y, Ma Z (2023). Phytopathology.

[3] Serpi M, Ferrari V, Pertusati F (2016). J Med Chem.

[4] Benner J P, Boehlendorf B G H, Kipps M R, Lambert N E P, Luck R, Molleyres L P, Neff S, Schuez T C, Stanley P D (2003). Biocidal compounds and their preparation. WO Pat. Appl..

[5] Li W, Csukai M, Corran A, Crowley P, Solomon P S, Oliver R P (2008). Pest Manage Sci.

[6] Winn M, Goss R J M, Kimura K-i, Bugg T D H (2010). Nat Prod Rep.

[7] Chen S, Kinney W A, Van Lanen S (2017). World J Microbiol Biotechnol.

[8] McErlean M, Liu X, Cui Z, Gust B, Van Lanen S G (2021). Nat Prod Rep.

[9] Zhang D, Miller M J (1999). Curr Pharm Des.

[10] Datta A, Merino P (2013). Synthetic Studies on Antifungal Peptidyl Nucleoside Antibiotics. Chemical Synthesis of Nucleoside Analogues.

[11] Danishefsky S, Hungate R (1986). J Am Chem Soc.

[12] Danishefsky S J, Hungate R, Schulte G (1988). J Am Chem Soc.

[13] Knapp S, Thakur V V, Madduru M R, Malolanarasimhan K, Morriello G J, Doss G A (2006). Org Lett.

[14] Fan S, Jiang T, Lv T, Liu J, Wang X (2023). Org Lett.

[15] Fan S, Jiang T, Siddique M N, Zhang L, Liu J, Wang X (2023). Org Lett.

[16] Hanessian S, Marcotte S, Machaalani R, Huang G (2003). Org Lett.

[17] Hanessian S, Huang G, Chenel C, Machaalani R, Loiseleur O (2005). J Org Chem.

[18] Hanessian S, Marcotte S, Machaalani R, Huang G, Pierron J, Loiseleur O (2006). Tetrahedron.

[19] Loiseleur O, Schneider H, Huang G, Machaalani R, Sellès P, Crowley P, Hanessian S (2006). Org Process Res Dev.

[20] Hanessian S, Ritson D J (2006). J Org Chem.

[21] Meng Y, Tao S, Wu X-Y, Huang S-H, Hong R (2023). Org Lett.

[22] Chen H, Lin Z, Meng Y, Li J, Huang S-H, Hong R (2023). Org Lett.

[23] Peng Y, Lin Z, Zhu L, Han S, Huang S-H, Hong R (2024). Chin J Chem.

[24] Liu Y, Zhao J, Hong R (2024). Org Lett.

[25] Wang Y, Lin Z, Huang S-H, Zhu L, Hong R (2025). Chin J Org Chem.

[26] Lin Z, Wu L, Yang S, Zhu L, Hong R, Huang S-H (2025). Org Lett.

[27] Liu Y, Yang C, Huang S-H, Hong R (2025). Eur J Org Chem.

[28] Fernandes R A, Fernandes R A (2018). Latent Functionality. Protecting-Group-Free Organic Synthesis: Improving Economy and Efficiency.

[29] Shing T K M, Leung G Y C (2002). Tetrahedron.

[30] Bhattacharjee A, Datta S, Chattopadhyay P, Ghoshal N, Kundu A P, Pal A, Mukhopadhyay R, Chowdhury S, Bhattacharjya A, Patra A (2003). Tetrahedron.

[31] Popik O, Grzeszczyk B, Staszewska-Krajewska O, Furman B, Chmielewski M (2020). Org Biomol Chem.

[32] Trifonova A, Földesi A, Dinya Z, Chattopadhyaya J (1999). Tetrahedron.

[33] Jana S (2007). Indian J Chem, Sect B: Org Chem Incl Med Chem.

[34] Zhong Y-L, Shing T K M (1997). J Org Chem.

[35] Padwa A (1976). Angew Chem, Int Ed Engl.

[36] Nair V, Suja T D (2007). Tetrahedron.

[37] Dupau P, Epple R, Thomas A A, Fokin V V, Sharpless K B (2002). Adv Synth Catal.

[38] Chatterjee A, Bhattacharya P K (2006). J Org Chem.

[39] Nagireddy J R, Tranmer G K, Carlson E, Tam W (2014). Beilstein J Org Chem.

[40] Dess D B, Martin J C (1983). J Org Chem.

[41] Fatima S, Zahoor A F, Khan S G, Naqvi S A R, Hussain S M, Nazeer U, Mansha A, Ahmad H, Chaudhry A R, Irfan A (2024). RSC Adv.

