# Supplementary material for: Rapid access to the core of malayamycin A by intramolecular dipolar cycloaddition
Source: Beilstein J Org Chem. 2025 Nov 17;21:2542–7. doi: 10.3762/bjoc.21.196 (PMC12642950; doi:10.3762/bjoc.21.196)

BEILSTEIN JOURNAL OF ORGANIC CHEMISTRY

Supporting Information

for

Rapid access to the core of malayamycin A by intramolecular dipolar cycloaddition

Yilin Liu, Yuchen Yang, Chen Yang, Sha-Hua Huang, Jian Jin and Ran Hong

Beilstein J. Org. Chem. **2025,** *21,* 2542–2547. doi:10.3762/bjoc.21.196

Experimental procedures and compound characterization data

License and Terms: This is a supporting information file under the terms of the Creative Commons Attribution License (https://creativecommons.org/licenses/by/4.0). Please note that the reuse, redistribution and reproduction in particular requires that the author(s) and source are credited and that individual graphics may be subject to special legal provisions.
The license is subject to the *Beilstein Journal of Organic Chemistry* terms and conditions: (https://www.beilstein-journals.org/bjoc/terms)

Contents

1. General procedures……………………………………………………………………......S2

2. Experimental procedures.…………………………………..………………………..S3

3. X-ray data for compound **19a**……………………………………….………......S12

4. ^{1}H NMR, ^{13}C NMR and 2D NMR spectra…………………………………………S13

1. General procedures

All reactions sensitive to air or moisture were conducted in oven-dried glassware fitted with rubber septa and magnetically stirred under argon atmosphere unless otherwise noted. All commercial grade reagents were used without further purification unless stated otherwise. Flash chromatography was carried out with 200-300 mesh silica gel (Silicycle® flash F60) with the indicated solvent systems.

Reactions were monitored by thin layer chromatography (TLC) supplied by Yantai Jiangyou Silicon Material Company (China). Visualization was accomplished with UV light or basic aqueous potassium permanganate ($KMnO_4$). NMR spectra were recorded on Varian mercury–400, Bruker AM–400, and Bruker AV–600 spectrometers and chemical shifts are reported in ppm using residual ^1H and ^{13}C signals of the solvent ($CDCl_3$: δ 7.26, 77.06 ppm; CD_3OD: δ 3.31, 49.00 ppm) as an internal standard. NMR experiments were all performed at 298 K. Signal patterns are indicated as: (s = singlet, br = broaden peak, d = doublet, t = triplet, q = quartet, m = multiplet). Structural assignments were made with additional information from gHMQC, gCOSY, gNOESY, and gHMBC experiments. Mass spectra were determined on a Shimadzu LCMS-2010EV(ESI) mass spectrometer or Agilent G6100 LC/MSD (ESI) single Quand mass spectrometer. High-resolution mass spectra (HRMS) were acquired through the National Center for Organic Mass Spectrometry in Shanghai on a Thermo Fisher Scientific LTQFT-Ultra mass spectrometer (mass analyzer type: Fourier transform ion cyclotron resonance (FT-ICR) with ESI (Electron Spray Ionization). Infrared (IR) spectra were recorded as thin films on KBr plates on a Perkin–Elmer 983 or Digital FTIR spectrometer and are reported in frequencies of absorption given in reciprocal centimeters (cm^{-1}).

2. Experimental procedure

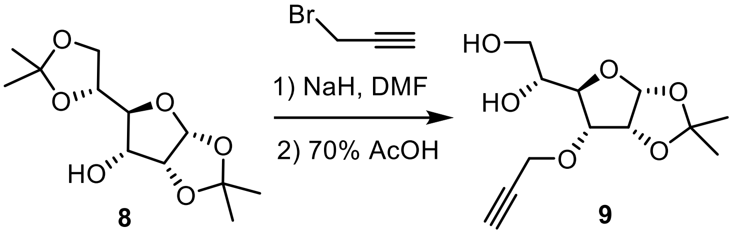

To a solution of **8** (2.6 g, 10 mmol) in DMF (25 mL) was added sodium hydride (480 mg, 12 mmol, 60% in mineral oil) at 0°C under nitrogen atmosphere. After stirring during 30 min, propargyl bromide (1.03 mL, 12 mmol) was added. The reaction was stirred at room temperature overnight. The reaction was then quenched with aqueous NH_4Cl and extracted with EA (3×50 mL). The combined organic layers were washed with brine (50 mL) and dried over Na_2SO_4, filtered and concentrated *in vacuo*. The residue was subjected to purification by flash column chromatography on silica gel (hexanes/EtOAc = 12/1) to give product as white solid (2.7 g, 90% yield). The obtained solid was dissolved in 70% AcOH (21 mL), and the reaction mixture was stirred at room temperature overnight, and concentrated under reduced pressure to obtain the crude product. The residue was subjected to purification by flash column chromatography on silica gel (DCM/ acetone = 5/1) to give **9**[1] as yellow oil (1.0 g, 77% yield, brsm 92%).

$[\alpha]_D^{25}$ = +96.5 (c = 0.5 in $CHCl_3$);

^{1}H NMR (400 MHz, $CDCl_3$) δ 5.79 (d, J = 3.5 Hz, 1H), 4.70 (t, J = 4.1 Hz, 1H), 4.39 (dd, J = 16.1, 2.2 Hz, 1H), 4.25 (dd, J = 16.1, 2.1 Hz, 1H), 4.18 (dd, J = 8.5, 4.1 Hz, 1H), 4.09–4.00 (m, 2H), 3.78–3.66 (m, 2H), 2.55 (t, J = 2.4 Hz, 1H), 1.57 (s, 3H), 1.35 (s, 3H).

HRMS-ESI (*m/z*): calcd. for $C_{12}H_{18}NaO_6$ $[M+Na]^+$: 281.0996; found: 281.0998

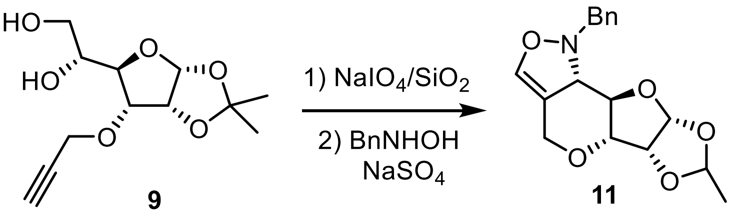

To a solution of **9** (140 mg, 0.54 mmol) in DCM (3 mL) was added $NaIO_4/SiO_2$ (20 %, 800 mg). The reaction mixture was stirred at room temperature for 30 min. The mixture was

[1] Jana, S. *Indian J. Chem. B.* **2007**, *45B*, 1648-1657.

filtered through a short plug of silica gel, washed with EtOAc (20 mL) and concentrated *in vacuo*. The crude reaction mixture was dissolved in toluene (5 mL), and then Na_2SO_4 (769 mg, 5.4 mmol) and BnNHOH (80 mg, 0.64 mmol) were added to the system. The reaction was stirred at room temperature until TLC showed complete consumption of the starting material. The mixture was filtered through a short plug of silica gel, washed with EtOAc (20 mL) and concentrated *in vacuo*. The residue was subjected to purification by flash column chromatography on silica gel (hexanes/EtOAc = 2/1) to give **11** as white solid (75 mg, 42% yield).

Mp 116–118 °C;

$[\alpha]_D^{25} = -285.0$ (c = 1.0 in CH_2Cl_2);

^{1}H NMR (600 MHz, $CDCl_3$): δ 7.42 (d, J = 7.3 Hz, 2H), 7.34 (t, J = 7.5 Hz, 2H), 7.29 (d, J = 7.3 Hz, 1H), 6.40 (s, 1H), 5.76 (d, J = 3.5 Hz, 1H), 4.66 (t, J = 3.8 Hz, 1H), 4.59 (d, J = 12.6 Hz, 1H), 4.28–4.15 (m, 2H), 4.12 (d, J = 8.9 Hz, 1H), 4.06 (dt, J = 12.5, 1.4 Hz, 1H), 3.96 (t, J = 9.0 Hz, 1H), 3.11 (dd, J = 9.4, 4.1 Hz, 1H), 1.61 (s, 3H), 1.35 (s, 3H).

^{13}C NMR (151MHz, $CDCl_3$): δ 139.5, 136.4, 129.2, 128.5, 127.7, 113.9, 104.5, 104.4, 79.9, 76.4, 74.0, 64.3, 64.1, 26.4, 26.3

FT-IR: ν (cm^{-1}): 2984, 2928, 2847, 1674, 1454, 1377, 1251, 1213, 1113, 1081, 1006, 862, 706, 637, 523.

HRMS-ESI (*m/z*): calcd. for $C_{18}H_{22}NO_5$ $[M+H]^+$: 332.1492; found: 332.1486

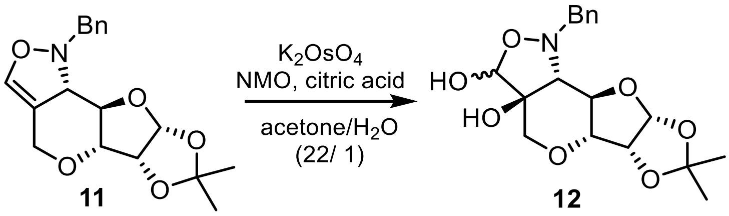

To a solution of **11** (600 mg, 1.8 mmol) in acetone/H_2O (9.4 mL, v/v 22:1) was added 4-Methylmorpholine *N*-oxide (421 mg, 3.6 mmol) and K_2OsO_4•$2H_2O$ (7.0 mg, 0.018 mmol). The reaction mixture was stirred at room temperature for 17 h. The reaction was then quenched with aqueous Na_2SO_3. The mixture was filtered through a short plug of silica gel, washed with EtOAc (50 mL) and concentrated *in vacuo*. The residue was subjected to

purification by flash column chromatography on silica gel (hexanes/EtOAc = 1/2) to give **12** as white solid (381 mg, 75% yield).

Mp 53–56 °C;

$[\alpha]_D^{25}$ = +39.6 (*c* = 0.8 in $CHCl_3$);

^{1}H NMR (400 MHz, $CDCl_3$) δ 7.42 (d, *J* = 7.4 Hz, 2H), 7.34–7.22 (m, 3H), 5.83 (d, *J* = 3.5 Hz, 1H), 5.27 (s, 1H), 4.65 (t, *J* = 3.8 Hz, 1H), 4.19 (s, 2H), 4.00 (d, *J* = 12.1 Hz, 1H), 3.66 (d, *J* = 11.9 Hz, 2H), 3.35 (d, *J* = 9.2 Hz, 2H), 1.62 (s, 3H), 1.37 (s, 3H).

^{13}C NMR (101 MHz, $CDCl_3$) δ 136.8, 129.6, 129.3, 128.5, 128.4, 127.8, 127.6, 114.1, 105.8, 75.9, 73.1, 71.6, 26.5, 26.3, 14.3

FT-IR: *v* (cm^{-1}): 3402, 1375, 1215, 1145, 1031, 962, 860, 744, 731, 621, 468, 408.

HRMS-ESI (*m/z*): calcd. for $C_{18}H_{24}NO_7$ $[M+H]^+$: 366.1547; found: 366.1547

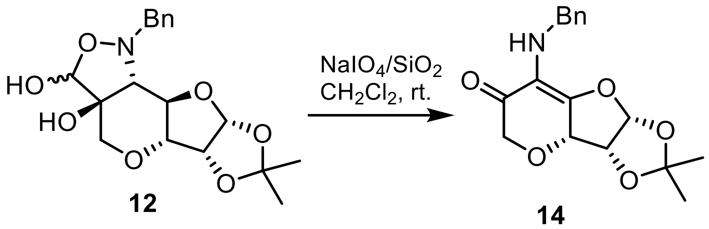

12 **14**

To a solution of **12** (10 mg, 0.027 mmol) in DCM (0.5 mL) was added $NaIO_4/SiO_2$ (20 %, 25 mg). The reaction mixture was stirred at room temperature for 20 min. The mixture was filtered through a short plug of silica gel, washed with EtOAc (20 mL) and concentrated *in vacuo*. The residue was subjected to purification by flash column chromatography on silica gel (hexanes/EtOAc = 1/1) to give **14** as white solid (6.0 mg, 60% yield).

Mp 140–142 °C;

$[\alpha]_D^{25}$ = −373.2 (*c* = 0.2 in $CHCl_3$);

^{1}H NMR (500 MHz, $CDCl_3$): δ 7.33–7.27 (m, 4H), 7.25–7.19 (m, 1H), 6.18–6.15 (m, 1H), 4.83–4.80 (m, 1H), 4.78–4.74 (m, 1H), 4.46–4.38 (m, 2H), 4.29 (d, *J* = 14.4 Hz, 1H), 4.23–4.14 (m, 1H), 4.04 (s, 1H), 1.45 (s, 3H), 1.44 (s, 3H).

^{13}C NMR (126 MHz, $CDCl_3$): δ 190.4, 150.3, 140.3, 128.6, 127.4, 127.2, 120.5, 116.9, 108.3, 79, 76.6, 71.7, 49.2, 27.8, 27.0

FT-IR: *v* (cm^{-1}):2987, 2841, 1693, 1627, 1475, 1375, 1205, 1118, 1035, 819, 783, 532.

HRMS-ESI (*m/z*): calcd. for $C_{17}H_{20}NO_5$ $[M+H]^+$: 318.1336; found: 318.1335

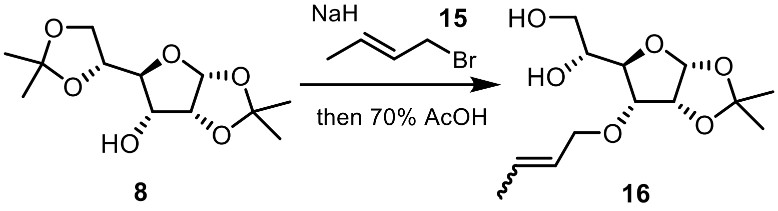

To a solution of **8** (3.9 g, 15 mmol) in DMF (30 mL) was added sodium hydride (720 mg, 18 mmol, 60% in mineral oil) at 0 °C under nitrogen atmosphere. After stirring during 10 min, crotyl bromide **15** (*E/Z* 5/1, 1.9 mL, 15.75 mmol) was added. The reaction was stirred at room temperature for additional 4.5 h. The reaction was then quenched with aqueous NH_4Cl and extracted with EA (3×50 mL). The combined organic layers were washed with brine (50 mL) and dried over Na_2SO_4, filtered and concentrated *in vacuo*. The residue was subjected to purification by flash column chromatography on silica gel (hexanes/EtOAc = 15/1) to give product as white solid (3.6 g, 78% yield). The obtained solid was dissolved in 70% AcOH (38 mL), and the reaction mixture was stirred at room temperature overnight, and concentrated under reduced pressure to obtain the crude product. The residue was subjected to purification by flash column chromatography on silica gel (hexanes/EtOAc = 1/1) to give **16**[2] as yellow oil (*E/Z* isomers, 3.3 g, 99% yield).

$[\alpha]_D^{25}$ = +110.2 (*c* = 1.0 in $CHCl_3$);

^{1}H NMR (400 MHz, $CDCl_3$) δ 5.83–5.65 (m, 2H), 5.65–5.53 (m, 1H), 4.62 (dt, *J* = 11.7, 4.0 Hz, 1H), 4.28 (dd, *J* = 11.3, 6.0 Hz, 0.3H), 4.20–4.08 (m, 1H), 4.06–3.93 (m, 2.8H), 3.92–3.85 (m, 1H), 3.72–3.60 (m, 2H), 2.87 (s, 1H), 2.81 (s, 1H), 1.69 (ddd, *J* = 13.3, 6.7, 1.6 Hz, 3H), 1.55 (s, 3H), 1.33 (s, 3H).

HRMS-ESI (*m/z*): calcd. For $C_{13}H_{22}NaO_6$ $[M+Na]^+$: 297.1309; found: 297.1302

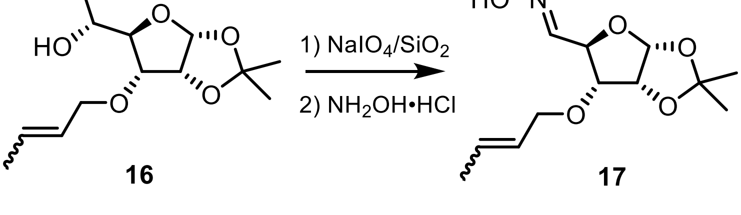

[2] Chatterjee, A.; Bhattacharya, P. K. *J. Org. Chem.* **2006**, *71*, 345-348.

To a solution of **16** (3.2 g, 11.6 mmol) in DCM (23 mL) was added $NaIO_4/SiO_2$ (20 %, 30 g). The reaction mixture was stirred at room temperature for 45 min. The mixture was filtered through a short plug of silica gel, washed with EtOAc (50 mL) and concentrated *in vacuo*. The crude reaction mixture was dissolved in ethanol (39 mL), and then $NaHCO_3$ (2.3 g, 39 mmol) and NH_2OH•HCl (2.2 g, 32.5 mmol) were added to the system. The reaction was stirred at 45 °C for 20 min. The mixture was filtered through a short plug of silica gel, washed with EtOAc (50 mL) and concentrated *in vacuo*. The residue was subjected to purification by flash column chromatography on silica gel (hexanes/EtOAc = 3/1) to give **17** as white solid (*E/Z* isomers, 2.29 g, 77% yield).

Mp 64–66 °C;

$[\alpha]_D^{25}$ = +40.3 (*c* = 1.0 in $CHCl_3$);

^{1}H NMR (400 MHz, $CDCl_3$) δ 7.80 (s, 1H), 7.39 (t, *J* = 6.4 Hz, 1H), 5.82 – 5.65 (m, 2H), 5.62–5.51 (m, 1H), 4.68–4.60 (m, 1H), 4.59–4.52 (m, 1H), 4.29–3.99 (m, 2H), 3.76 (dt, *J* = 8.7, 4.3 Hz, 1H), 1.75–1.65 (m, 3H), 1.59 (s, 3H), 1.36 (s, 3H).

^{13}C NMR (101 MHz, $CDCl_3$): δ 148.9, 131.0, 127.3, 114.1, 104.2, 80.1, 77.9, 75.9, 71.7, 26.9 (2 carbons), 17.9

FT-IR: *v* (cm^{-1}): 3435, 2983, 1377, 1251, 1213, 1166, 1114, 1082, 1066, 1018, 1001, 974, 970, 869, 651, 522.

HRMS-ESI (*m/z*): calcd. For $C_{12}H_{19}NNaO_5$ $[M+Na]^+$: 280.1155; found: 280.1159

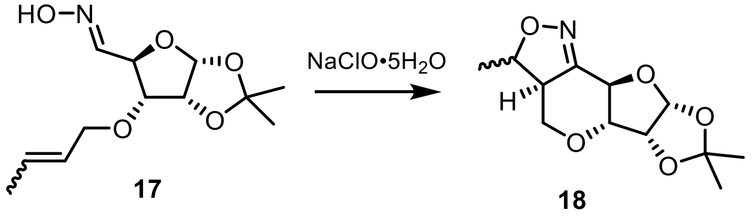

To a solution of **17** (280 mg, 1.09 mmol) in DCM (55 mL) was added NaOCl•$5H_2O$ (360 mg, 2.18 mmol). The reaction mixture was stirred at room temperature overnight. The reaction was then quenched with anhydrous $Na_2S_2O_3$ and extracted with EA (3×50 mL). The combined organic layers were washed with brine (50 mL) and dried over Na_2SO_4, filtered and concentrated *in vacuo*. The residue was subjected to purification by flash column

chromatography on silica gel (hexanes/EtOAc = 1/1) to give **18** as white solid (*α/β*-methyl isomers, 220 mg, 79% yield, *dr* = 4:1).

Mp 197–201 °C;

$[\alpha]_D^{25}$ = −213.4 (*c* = 1.0 in $CHCl_3$);

^{1}H NMR (400 MHz, $CDCl_3$): δ 5.87–5.84 (m, 1H), 4.83–4.73 (m, 0.26H), 4.73–4.68 (m, 1.26H), 4.50 (d, *J* = 9.8 Hz, 0.25H), 4.45 (d, *J* = 9.7 Hz, 1H), 4.34 (dd, *J* = 10.9, 7.3 Hz, 1H), 4.25–4.15 (m, 1.25H), 3.65 (t, *J* = 11.1 Hz, 0.25H), 3.45 (t, *J* = 10.7 Hz, 1H), 3.43–3.34 (m, 0.25H), 3.17 (dd, *J* = 9.8, 3.9 Hz, 1H), 3.13 (dd, 0.25H), 3.02 (td, *J* = 10.0, 7.6 Hz, 1H), 1.54 (s, 3.8H), 1.39 (d, *J* = 6.2 Hz, 3H), 1.31 (s, 3.8H), 1.17 (d, *J* = 6.7 Hz, 0.8H).

^{13}C NMR (101 MHz, $CDCl_3$): δ 156.1, 155.1, 114.2, 105.7, 105.6, 83.3, 83.3, 79.0, 77.4, 76.3, 76.0, 75.9, 73.4, 71.5, 71.4, 69.6, 53.8, 49.4, 26.2, 26.0, 19.6, 15.4

FT-IR: *v* (cm^{-1}): 2983, 2879, 1377, 1251, 1211, 1112, 1001, 931, 862, 846, 406.

HRMS-ESI (*m/z*): calcd. for $C_{12}H_{18}NO_5$ $[M+Na]^+$: 256.1179; found: 256.1189

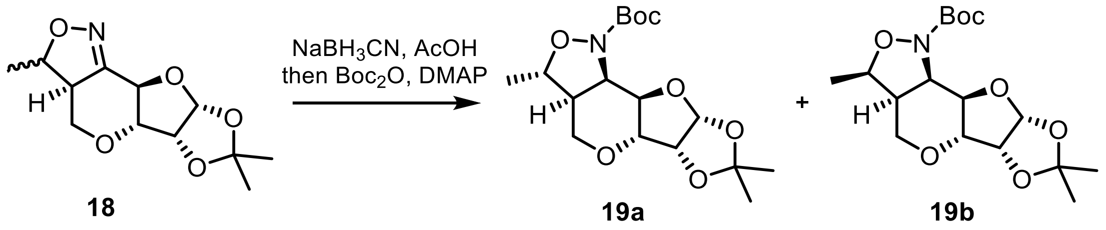

To a solution of **18** (255 mg, 1 mmol, *dr* = 4:1) in DCM (10 mL) and AcOH (10 mL) was added $NaBH_3CN$ (252 mg, 4 mmol). The reaction mixture was stirred at room temperature for 2 h. TLC analysis indicated incomplete conversion of the starting material. The reaction was quenched with dropwise addition of NaOH solution to adjust the pH to 7, and extracted with EA (3×30 mL). The combined organic layers were washed with brine (50 mL) and dried over Na_2SO_4, filtered and concentrated *in vacuo*. The residue was subjected to purification by flash column chromatography on silica gel (hexanes/EtOAc = 1/1) to give product as yellow oil, which was used directly in the next step without further purification. To a solution of obtained crude oil (250 mg, 0.9 mmol) in DCM (3 mL) was added Boc_2O (1.5 mL, 6.5 mmol) and DMAP (22 mg, 0.09 mmol). The reaction mixture was stirred at room temperature for 3h. The reaction mixture was subjected to purification by flash

chromatography on silica gel (hexanes/EtOAc = 3/1) to afford **19a** and **19b** (207 mg, total 58% yield, *dr* = 4:1) as white solid.

19a (major):

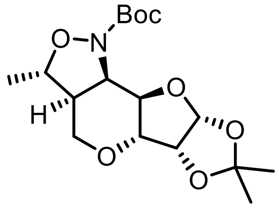

Mp 198–201 °C;

$[\alpha]_D^{25}$ = −185.2 (*c* = 0.5 in $CHCl_3$);

^{1}H NMR (600 MHz, $CDCl_3$): δ 5.81 (d, *J* = 3.4 Hz, 1H), 4.70 (dd, *J* = 6.1, 4.2 Hz, 1H), 4.61 (t, *J* = 3.9 Hz, 1H), 4.20 (dd, *J* = 11.7, 7.0 Hz, 1H), 4.10 (dd, *J* = 9.9, 4.0 Hz, 1H), 3.93 (q, *J* = 6.4 Hz, 1H), 3.58 (dd, *J* = 11.7, 9.8 Hz, 1H), 3.46 (dd, *J* = 9.9, 4.4 Hz, 1H), 2.69 (dt, *J* = 9.7, 6.7 Hz, 1H), 1.57 (s, 3H), 1.50 (s, 9H), 1.34 (s, 3H), 1.16 (d, *J* = 6.4 Hz, 3H).

^{13}C NMR (151 MHz, $CDCl_3$): δ 159.2, 113.4, 105.0, 82.4, 78.9, 76.5, 74.6, 73.4, 69.6, 57.8, 47.1, 28.3, 26.5, 26.2, 17.2

FT-IR: *v* (cm^{-1}): 2983, 2879, 1377, 1211, 1112, 1068, 1003, 863, 838, 509.

HRMS-ESI (*m/z*): calcd. for $C_{17}H_{27}NNaO_7$ $[M+Na]^+$: 380.1680; found: 380.1682

19b (minor):

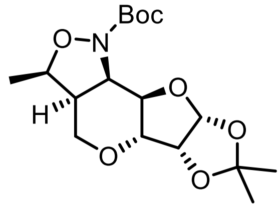

Mp 80–82 °C;

$[\alpha]_D^{25}$ = +73.6 (*c* = 0.1 in $CHCl_3$);

^{1}H NMR (600 MHz, $CDCl_3$) δ 5.81 (d, *J* = 3.4 Hz, 1H), 4.72 (dd, *J* = 6.6, 4.1 Hz, 1H), 4.60 (t, *J* = 3.9 Hz, 1H), 4.17 (dd, *J* = 10.1, 4.1 Hz, 1H), 4.11–4.01 (m, 2H), 3.83 (dd, *J* = 12.0, 8.3 Hz, 1H), 3.53 (dd, *J* = 10.1, 4.3 Hz, 1H), 2.79–2.72 (m, 1H), 1.59 (s, 3H), 1.50 (s, 9H), 1.35 (s, 3H), 1.30 (d, *J* = 6.4 Hz, 3H).

^{13}C NMR (151 MHz, $CDCl_3$): δ 158.4, 113.3, 105.0, 82.6, 77.8, 76.7, 74.4, 73.1, 65.5, 44.3, 28.3, 26.5, 26.1, 12.2

FT-IR: *v* (cm^{-1}): 3354, 2980, 2920, 2864, 1720, 1664, 1367, 1255, 1244, 1159, 1085, 1045,

1008, 856, 435.

HRMS-ESI (*m/z*): calcd. For $C_{17}H_{27}NNaO_7$ $[M+Na]^+$: 380.1680; found: 380.1689

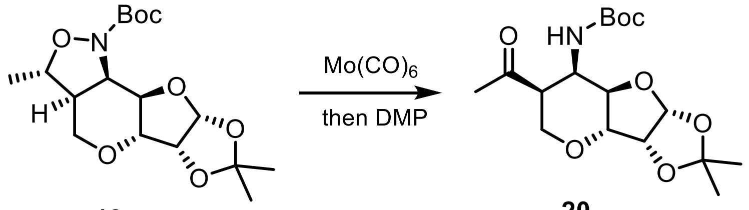

To a solution of **19a** (67 mg, 0.18 mmol) in CH_3CN (14.4 mL) and H_2O (3.6 mL) was added $Mo(CO)_6$ (238 mg, 0.9 mmol). The reaction mixture was stirred at 135 °C for 2.5 h. The reaction mixture was cooled to room temperature, filtered through a short plug of silica gel and concentrated *in vacuo*. The crude was used directly in the next step without further purification. To a solution of crude (45 mg, 0.12 mmol) in DCM (5 mL) was added DMP (80 mg, 0.19 mmol). The reaction mixture was stirred at room temperature until TLC analysis indicated complete conversion of the starting material. The reaction mixture was added aqueous $NaHCO_3$ to adjust the pH to 7 and then quenched with aqueous $Na_2S_2O_3$, extracted with EA (3×20 mL). The combined organic layers were washed with brine (20 mL) and dried over Na_2SO_4, filtered and concentrated *in vacuo*. The residue was subjected to purification by flash column chromatography on silica gel (hexanes/EtOAc = 5/1) to give **20** as white solid (32 mg, 50% yield).

Mp 180–184 °C;

$[\alpha]_D^{25}$ = −40.0 (*c* = 0.7 in $CHCl_3$);

^{1}H NMR (400 MHz, $CDCl_3$): δ 5.80 (d, *J* = 3.4 Hz, 1H), 5.04–4.91 (m, 1H), 4.72–4.61 (m, 2H), 4.04 (dd, *J* = 12.8, 4.7 Hz, 1H), 3.98 (dd, *J* = 10.1, 4.0 Hz, 1H), 3.81 (t, *J* = 12.3 Hz, 1H), 3.23 (dd, *J* = 10.0, 3.9 Hz, 1H), 2.97 (dt, *J* = 11.7, 4.2 Hz, 1H), 2.27 (s, 3H), 1.59 (s, 3H), 1.41 (s, 9H), 1.35 (s, 3H).

^{13}C NMR (101 MHz, $CDCl_3$): δ 155.6, 114.0, 105.0, 80.6, 77.4, 76.3, 75.6, 73.1, 66.0, 51.3, 48.4, 29.2, 28.3, 26.4, 26.2

FT-IR: *v* (cm^{-1}): 3398, 2970, 1510, 1365, 1244, 1155, 1014, 983, 960, 852.

HRMS-ESI (*m/z*): calcd. For $C_{17}H_{27}NNaO_7$ $[M+Na]^+$: 380.1680; found: 380.1687

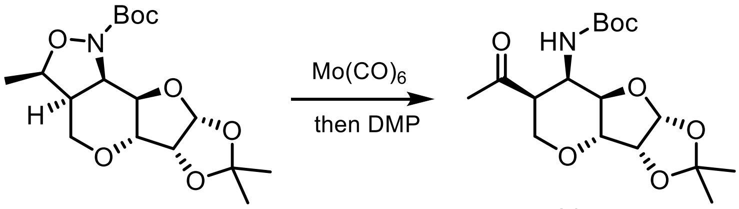

19b **20**

To a solution of **19b** (30 mg, 0.08 mmol) in CH_3CN (6.3 mL) and H_2O (1.6 mL) was added $Mo(CO)_6$ (105 mg, 0.40 mmol). The reaction mixture was stirred at 135 °C for 2.5 h. The reaction mixture was cooled to room temperature, filtered through a short plug of silica gel and concentrated *in vacuo*. The crude was used directly in the next step without further purification. To a solution of crude (18 mg, 0.05 mmol) in DCM (3 mL) was added DMP (35 mg, 0.08 mmol). The reaction mixture was stirred at room temperature until TLC analysis indicated complete conversion of the starting material. The reaction mixture was added aqueous $NaHCO_3$ to adjust the pH to 7 and then quenched with aqueous $Na_2S_2O_3$, extracted with EA (3×10 mL). The combined organic layers were washed with brine (20 mL) and dried over Na_2SO_4, filtered and concentrated *in vacuo*. The residue was subjected to purification by flash column chromatography on silica gel (hexanes/EtOAc = 5/1) to give **20** as white solid (15 mg, 53% yield). The spectra of **19b** are identical with the above known compound **20** derived from **19a**.

3. X-ray structure of 19a (CCDC 2483598)

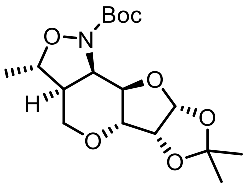

19a

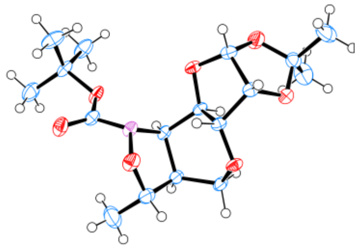

Identification code	mj25319_0m	
Empirical formula	$C_{17}H_{27}NO_7$	
Formula weight	357.39 g/mol	
Temperature	170.00 K	
Wavelength	Ga Kα (λ = 1.34139 Å)	
Crystal system	Orthorhombic	
Space group	P212121	
Unit cell dimensions	a = 8.97210(10) Å	a= 90°.
	b = 11.7715(2) Å	b= 90°.
	c = 17.2522(2) Å	g = 90°.
Volume	1822.09(4) Å3	
Z	4	
Density (calculated)	1.303 Mg/m3	
Absorption coefficient	0.559 mm-1	
F(000)	768	
Crystal size	0.17 x 0.17 x 0.05 mm3	
Theta range for data collection	4.459 to 63.446°.	
Index ranges	-11<=h<=11, -15<=k<=15, -22<=l<=23	
Reflections collected	20917	
Independent reflections	4494 [R(int) = 0.0359]	
Completeness to theta = 53.594°	99.3 %	
Absorption correction	Semi-empirical from equivalents	
Max. and min. transmission	0.7523 and 0.5836	
Refinement method	Full-matrix least-squares on F2	
Data / restraints / parameters	4494 / 0 / 232	
Goodness-of-fit on F2	1.039	
Final R indices [I>2sigma(I)]	R1 = 0.0302, wR2 = 0.0813	
R indices (all data)	R1 = 0.0306, wR2 = 0.0816	
Absolute structure parameter	0.03(4)	
Extinction coefficient	n/a	
Largest diff. peak and hole	0.265 and -0.144 e.Å-3	

4. ^{1}H NMR, ^{13}C NMR and 2D-NMR spectra

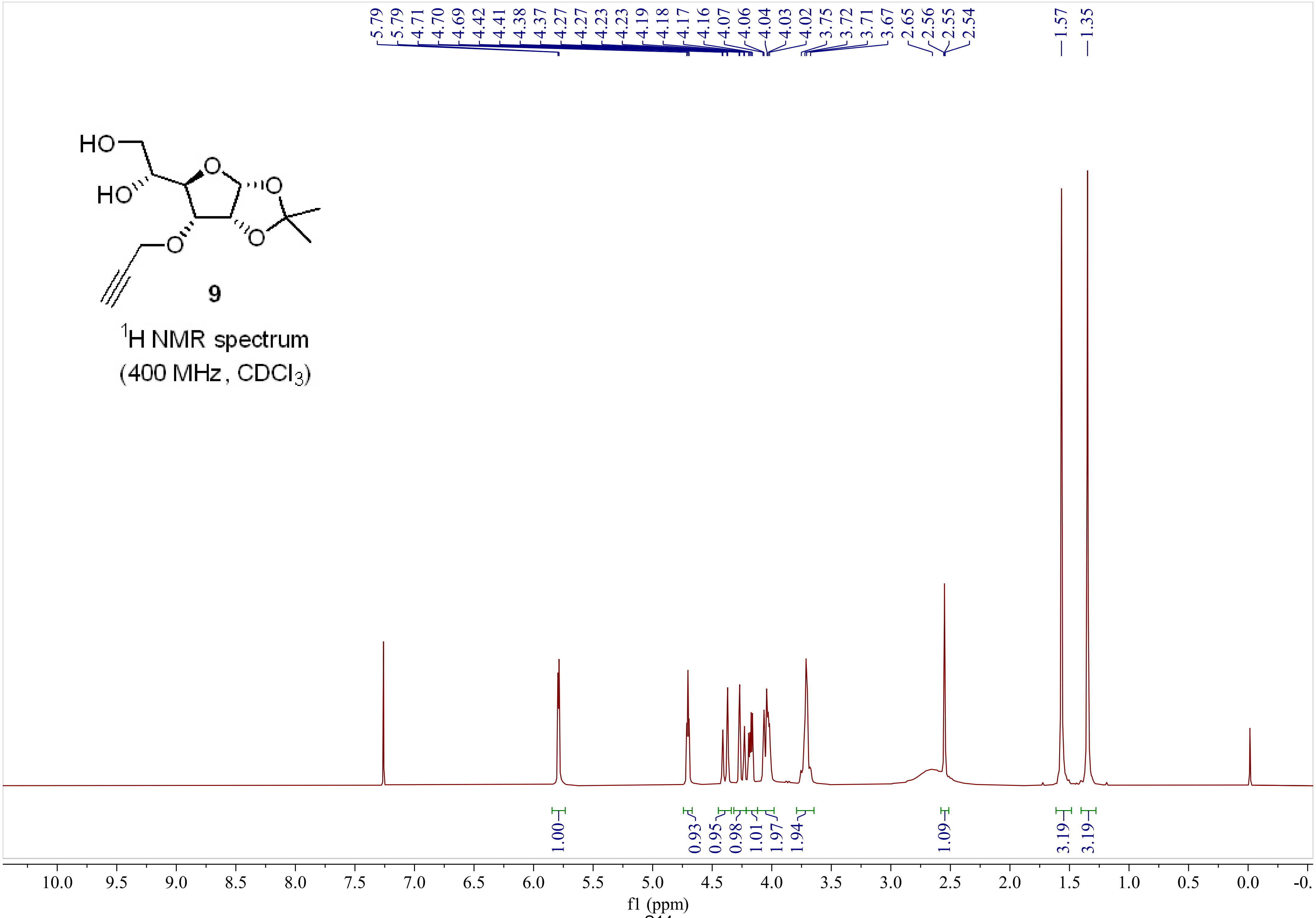

HO
HO
O
O
O
O
9
1H NMR spectrum
(400 MHz, CDCl3)
5.79 5.79 4.71 4.70 4.69 4.42 4.41 4.38 4.37 4.27 4.27 4.23 4.23 4.19 4.18 4.17 4.16 4.07 4.06 4.04 4.03 4.02 3.75 3.72 3.71 3.67 2.65 2.56 2.55 2.54 1.57 1.35
1.00 0.93 0.95 0.98 1.01 1.97 1.94 1.09 3.19 3.19
10.0 9.5 9.0 8.5 8.0 7.5 7.0 6.5 6.0 5.5 5.0 4.5 4.0 3.5 3.0 2.5 2.0 1.5 1.0 0.5 0.0 -0.
f1 (ppm)

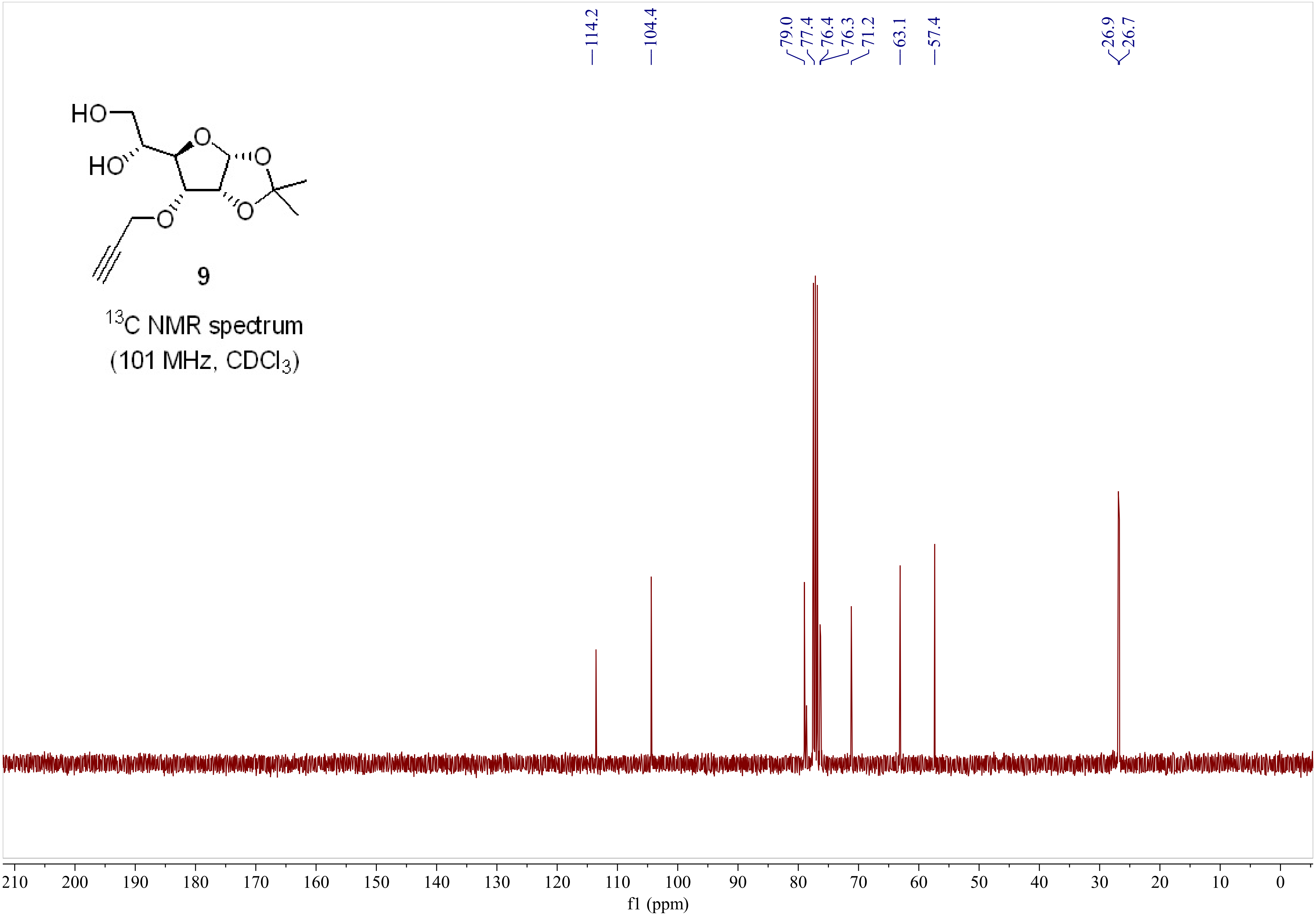
114.2
104.4
79.0
77.4
76.4
76.3
71.2
63.1
57.4
26.9
26.7
HO
HO
O
O
O
O
9
^{13}C NMR spectrum
(101 MHz, $CDCl_3$)
210 200 190 180 170 160 150 140 130 120 110 100 90 80 70 60 50 40 30 20 10 0
f1 (ppm)

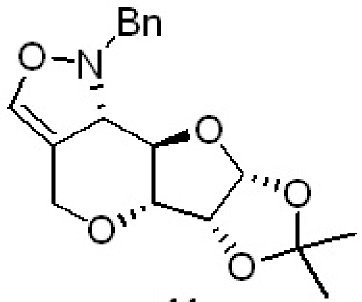

11

^{1}H NMR spectrum

(600 MHz, $CDCl_3$)

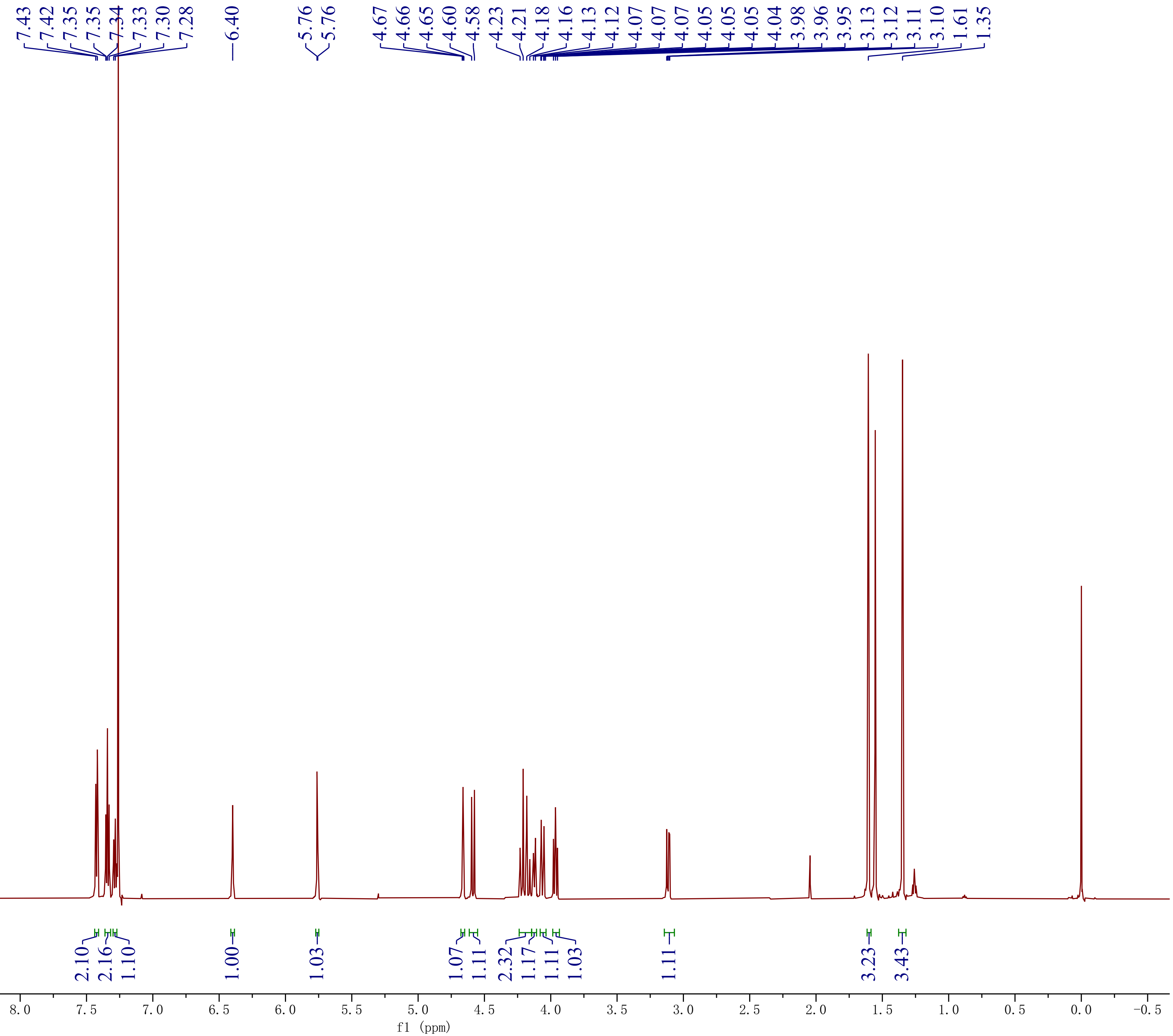

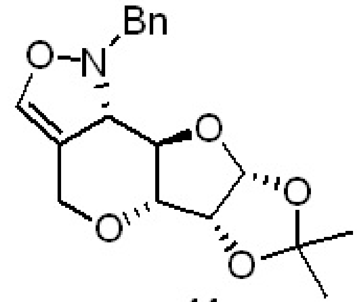

11

^{13}C NMR spectrum
(151 MHz, $CDCl_3$)

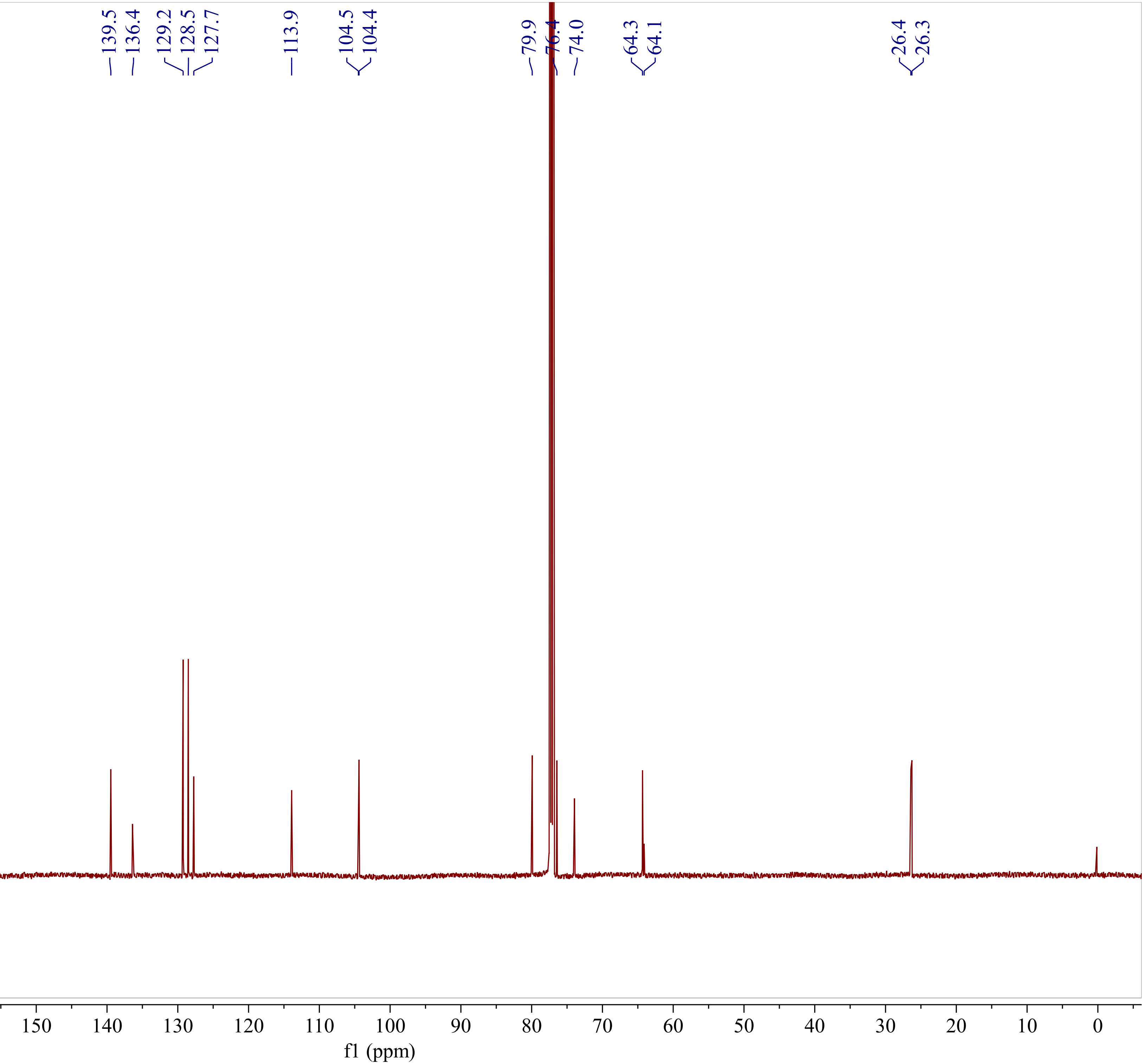

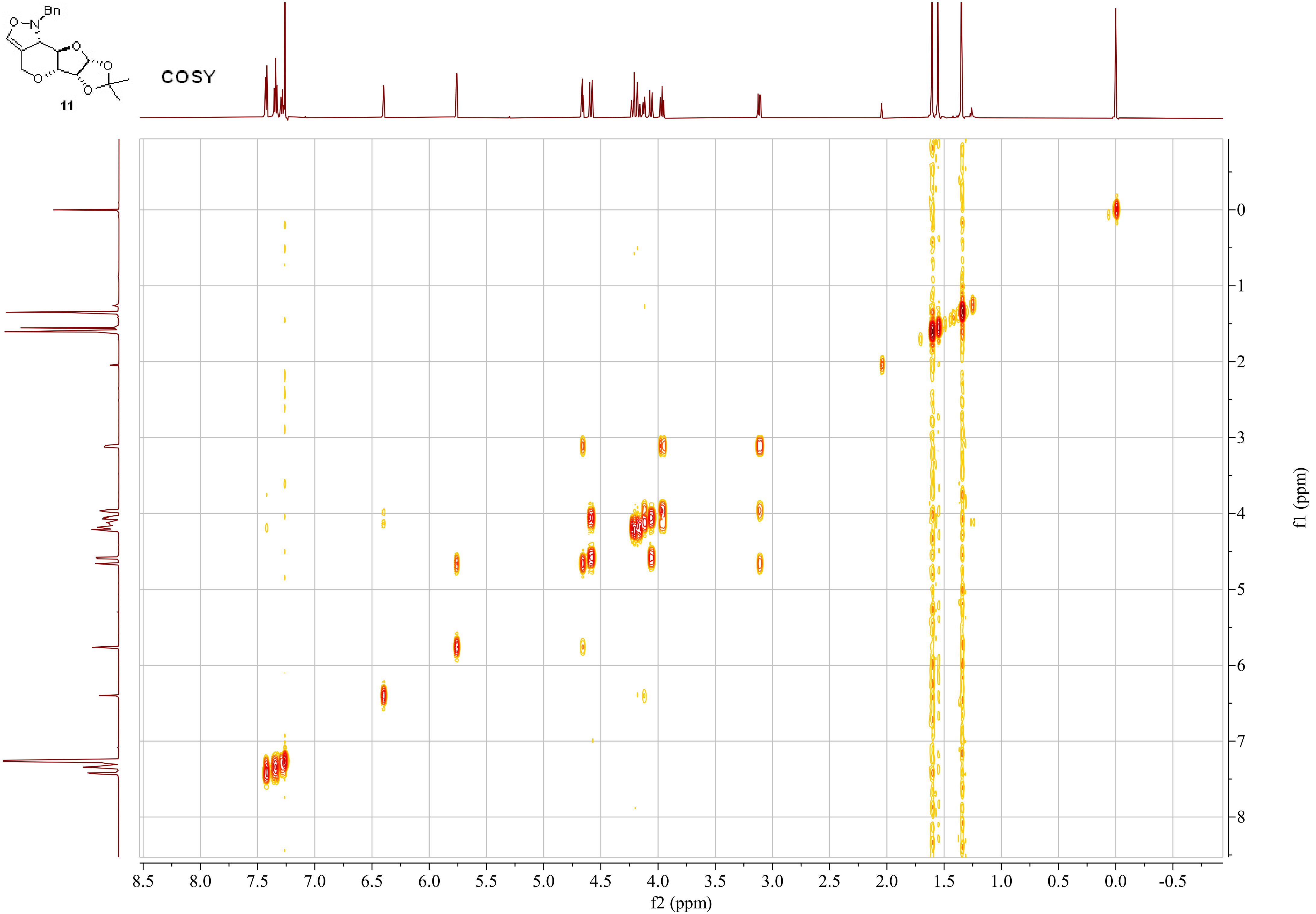
Bn
11
COSY
f2 (ppm)
f1 (ppm)

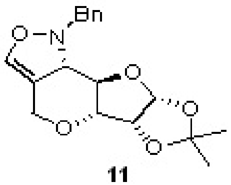

11

HMQC

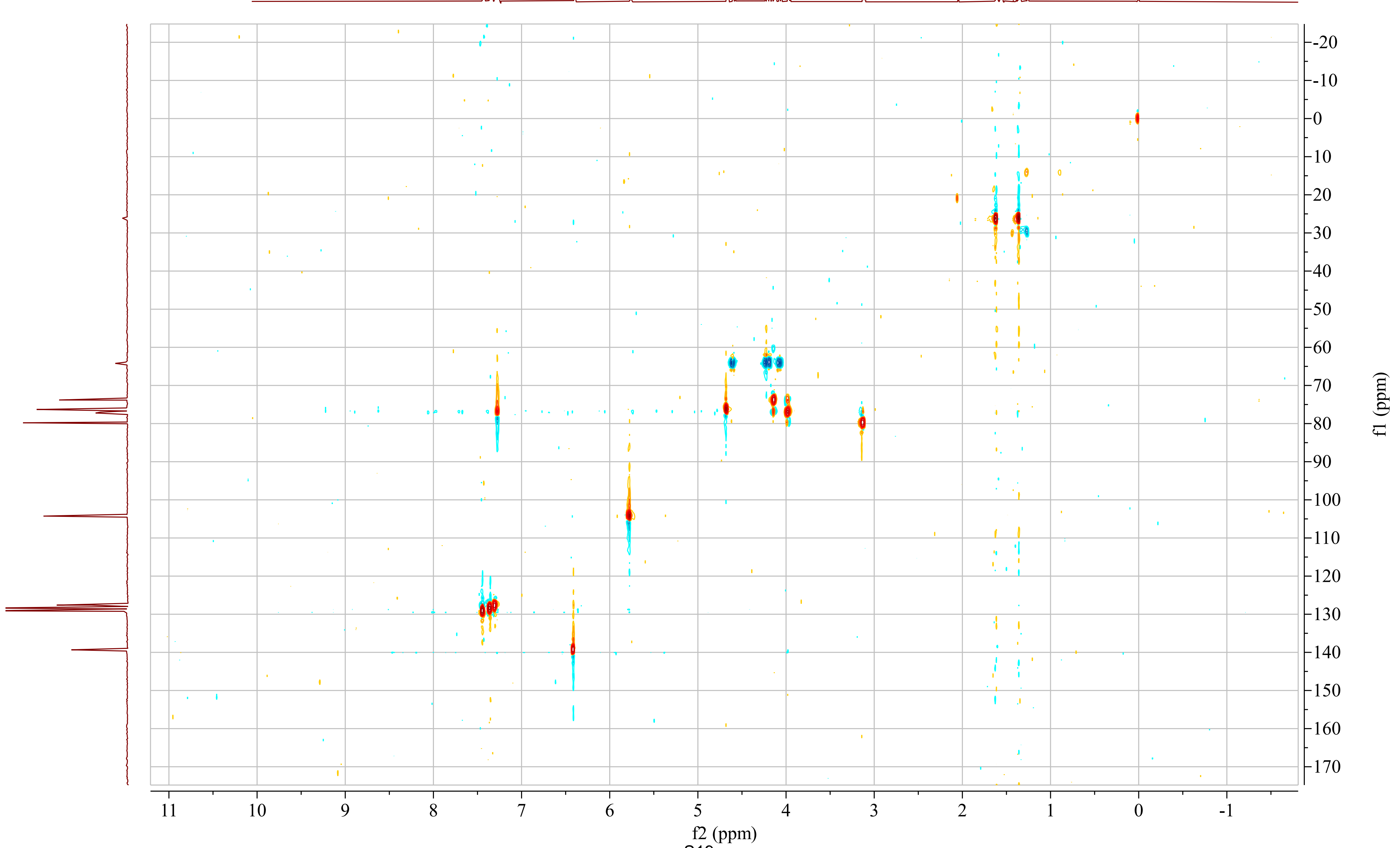

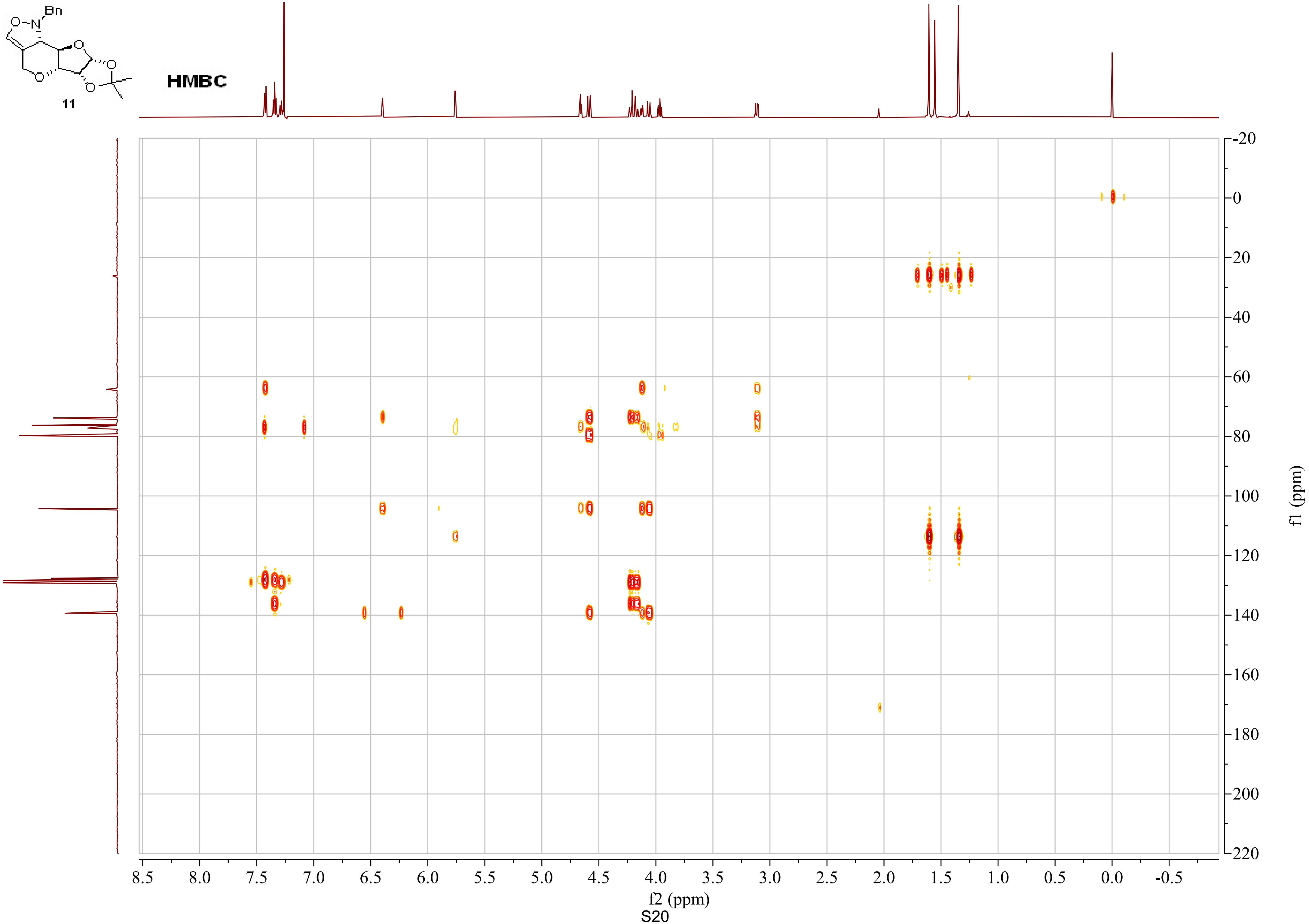
Bn
11
HMBC
f2 (ppm)
f1 (ppm)

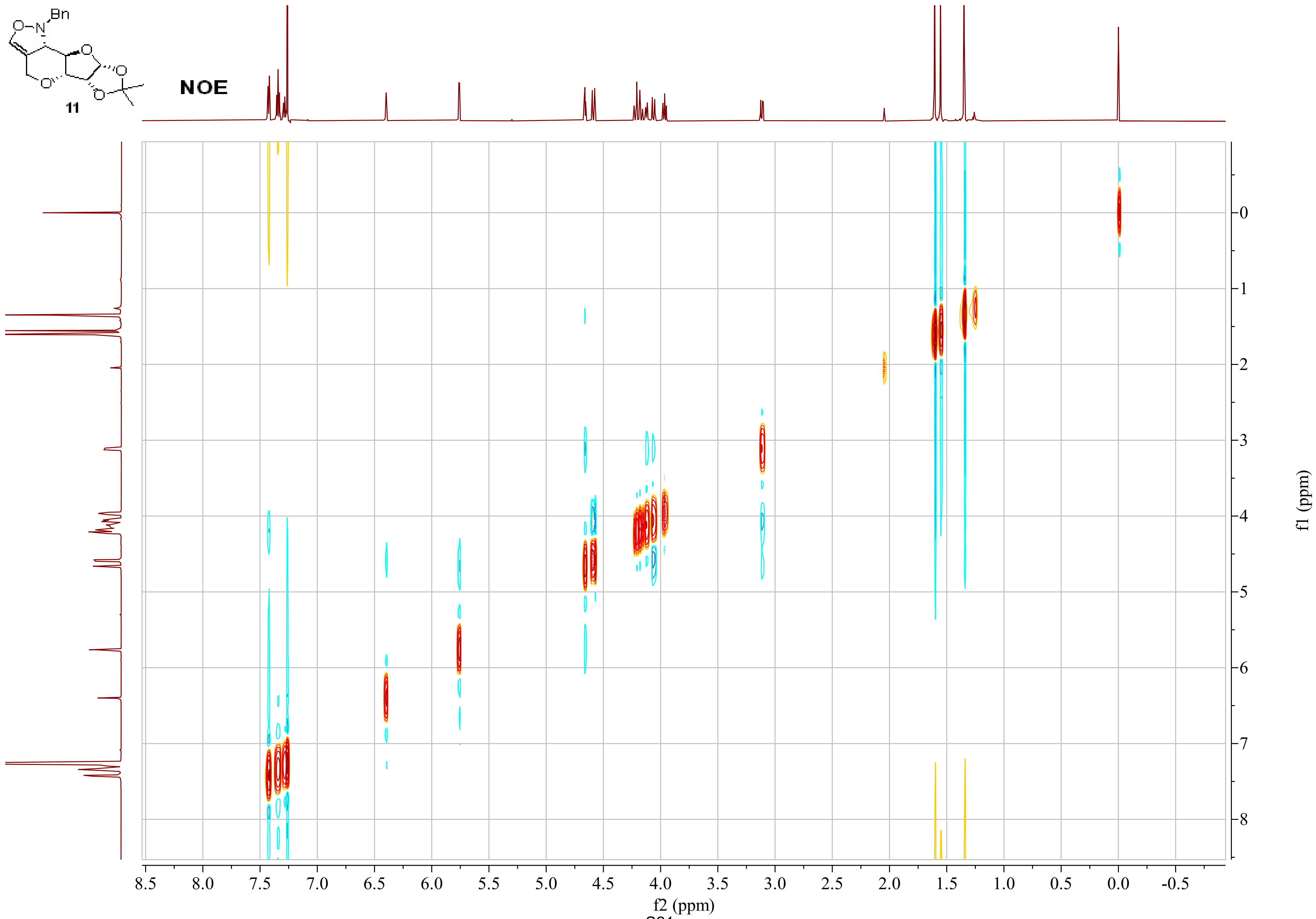

Bn
11
NOE
f1 (ppm)
f2 (ppm)

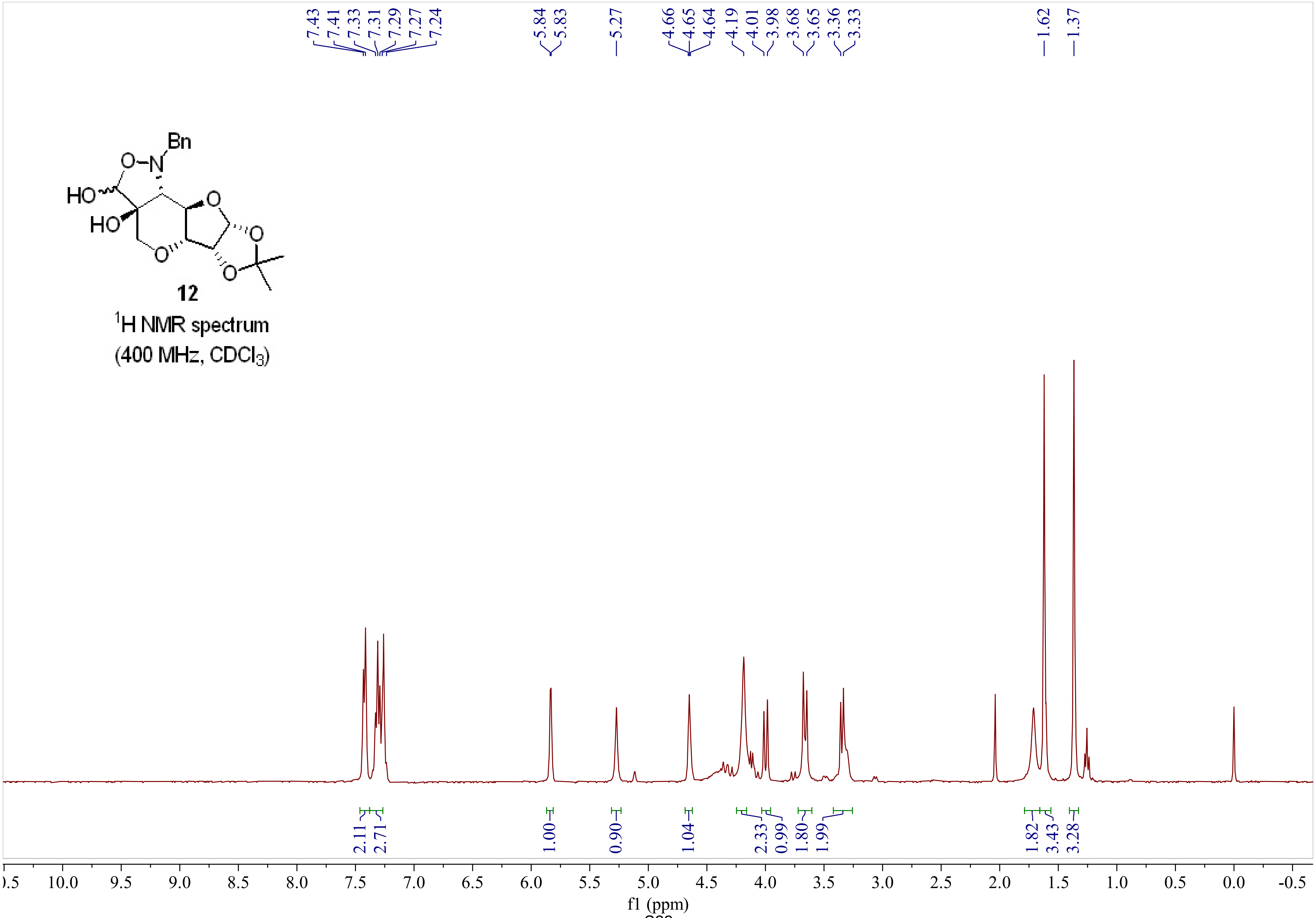

Bn
HO
HO
12
¹H NMR spectrum
(400 MHz, CDCl3)
7.43 7.41 7.33 7.31 7.29 7.27 7.24
5.84 5.83
5.27
4.66 4.65 4.64 4.19 4.01 3.98 3.68 3.65 3.36 3.33
1.62 1.37
2.11 2.71 1.00 0.90 1.04 2.33 0.99 1.80 1.99 1.82 3.43 3.28
10.0 9.5 9.0 8.5 8.0 7.5 7.0 6.5 6.0 5.5 5.0 4.5 4.0 3.5 3.0 2.5 2.0 1.5 1.0 0.5 0.0 -0.5
f1 (ppm)

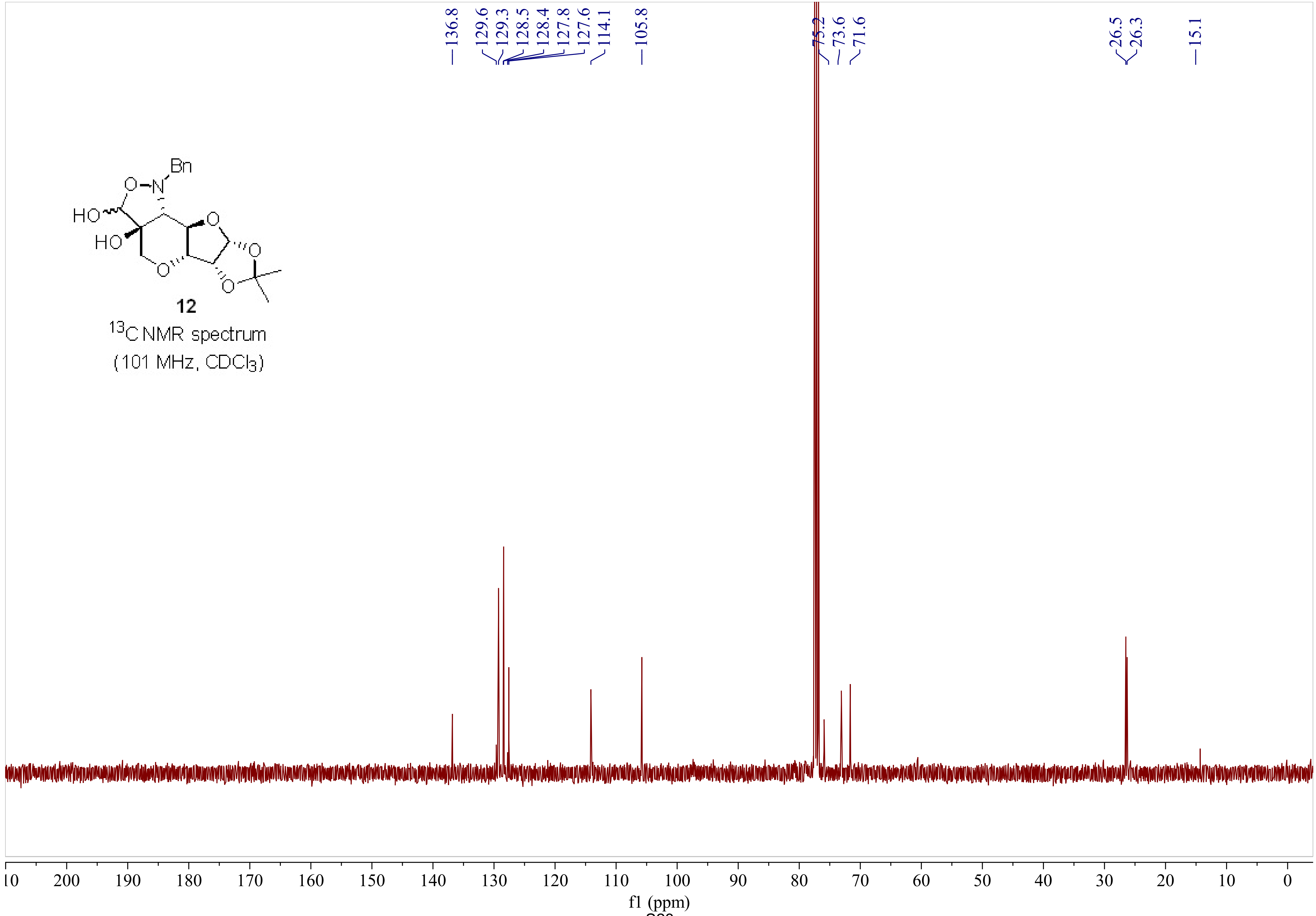
136.8
129.6
129.3
128.5
128.4
127.8
127.6
114.1
105.8
75.2
73.6
71.6
26.5
26.3
15.1
Bn
O
N
HO
HO
O
O
O
O
12
13C NMR spectrum
(101 MHz, CDCl3)
f1 (ppm)

14

^{1}H NMR spectrum

(500 MHz, $CDCl_3$)

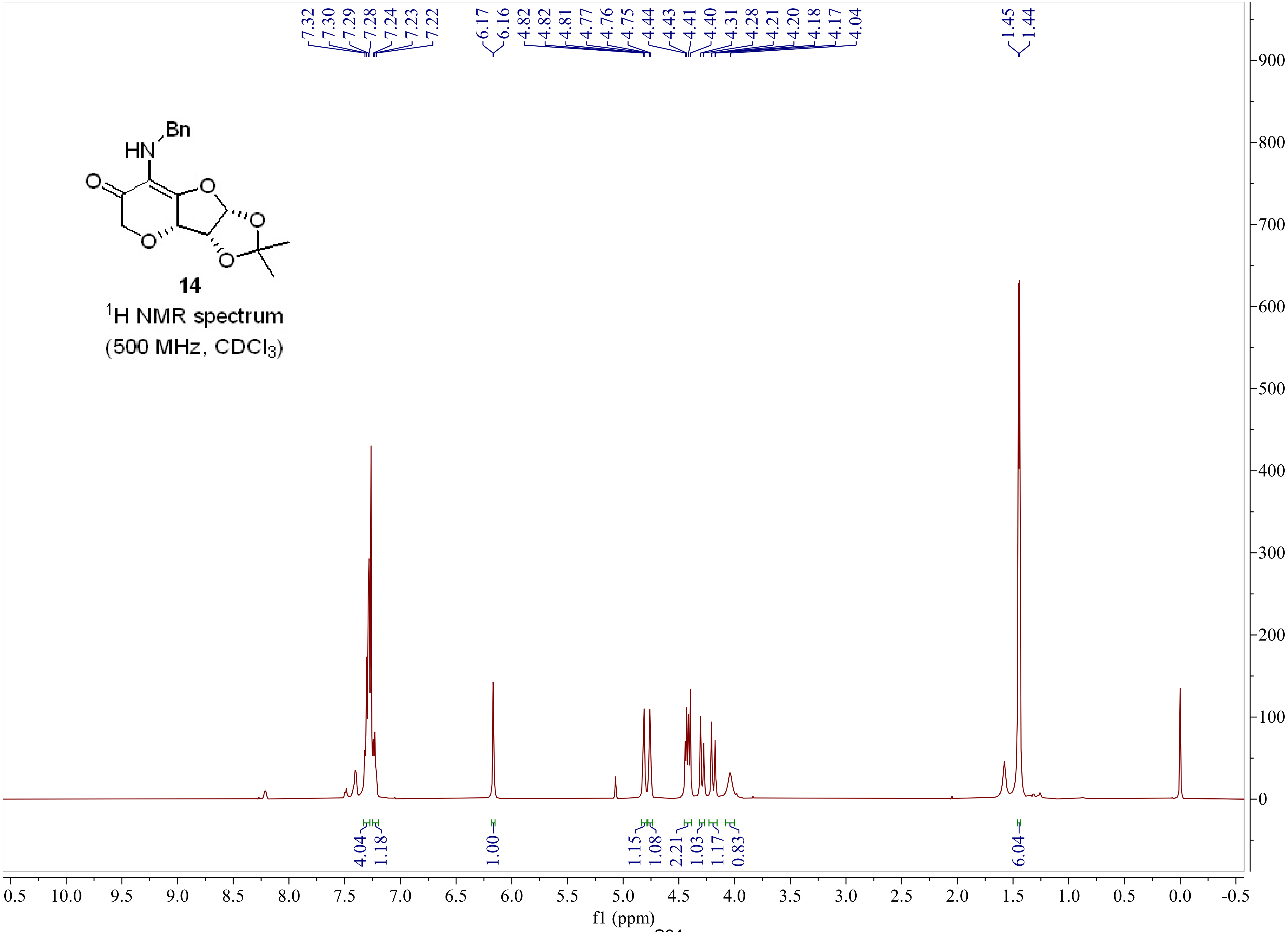

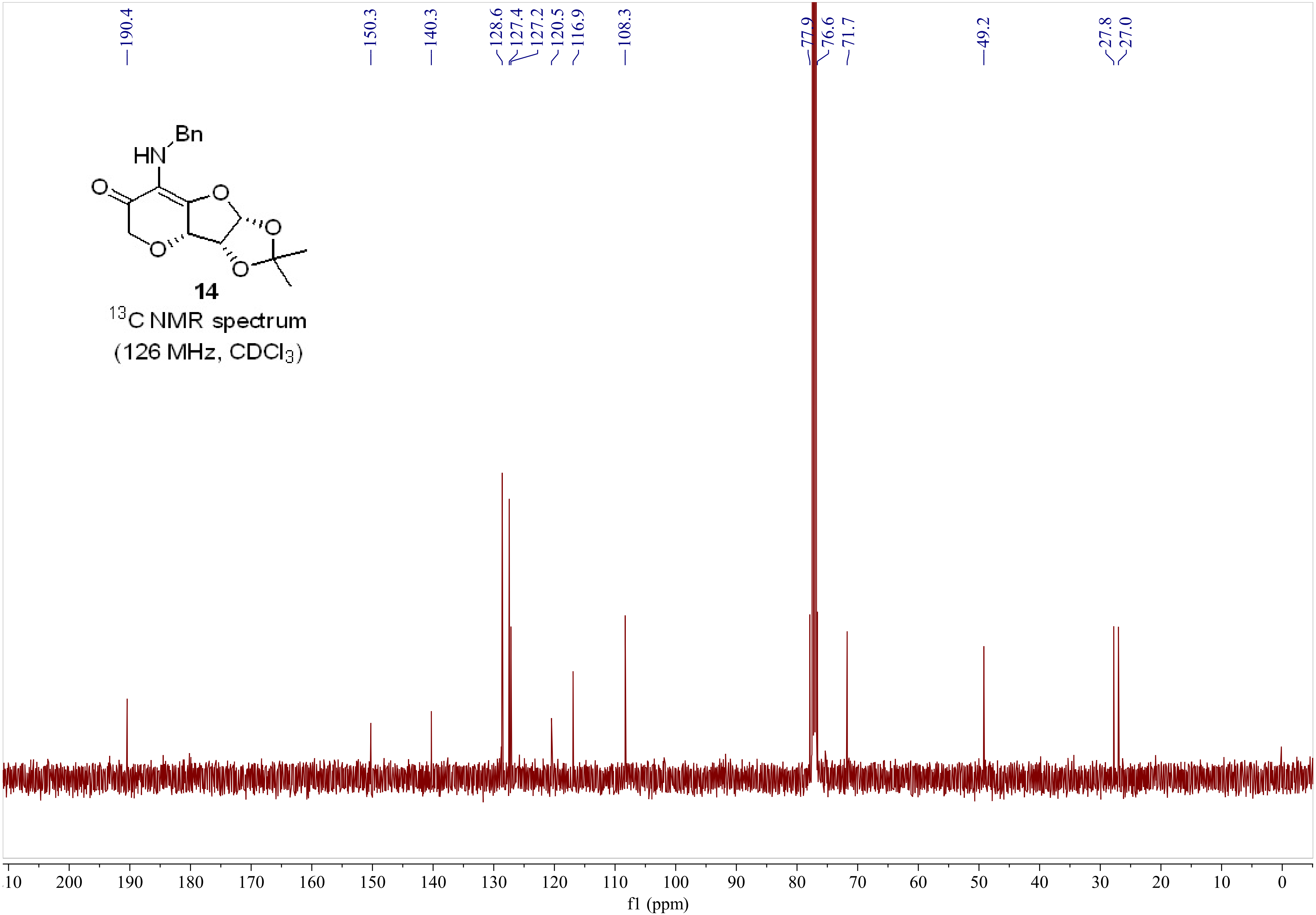

190.4
150.3
140.3
128.6
127.4
127.2
120.5
116.9
108.3
77.9
76.6
71.7
49.2
27.8
27.0
Bn
HN
O
14
^{13}C NMR spectrum
(126 MHz, $CDCl_3$)
f1 (ppm)

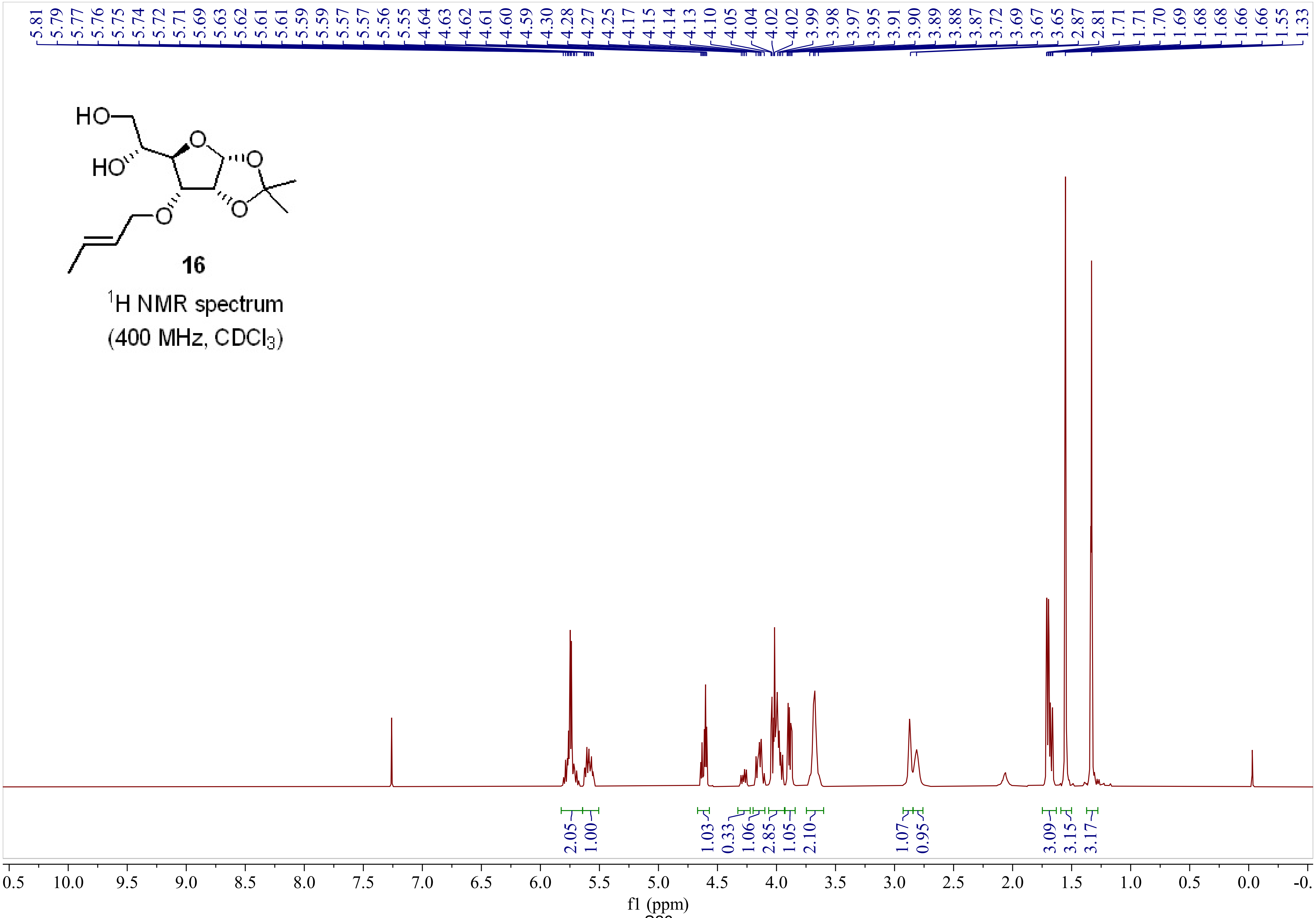

HO
HO
O
O
O
O
16
1H NMR spectrum
(400 MHz, CDCl3)
f1 (ppm)

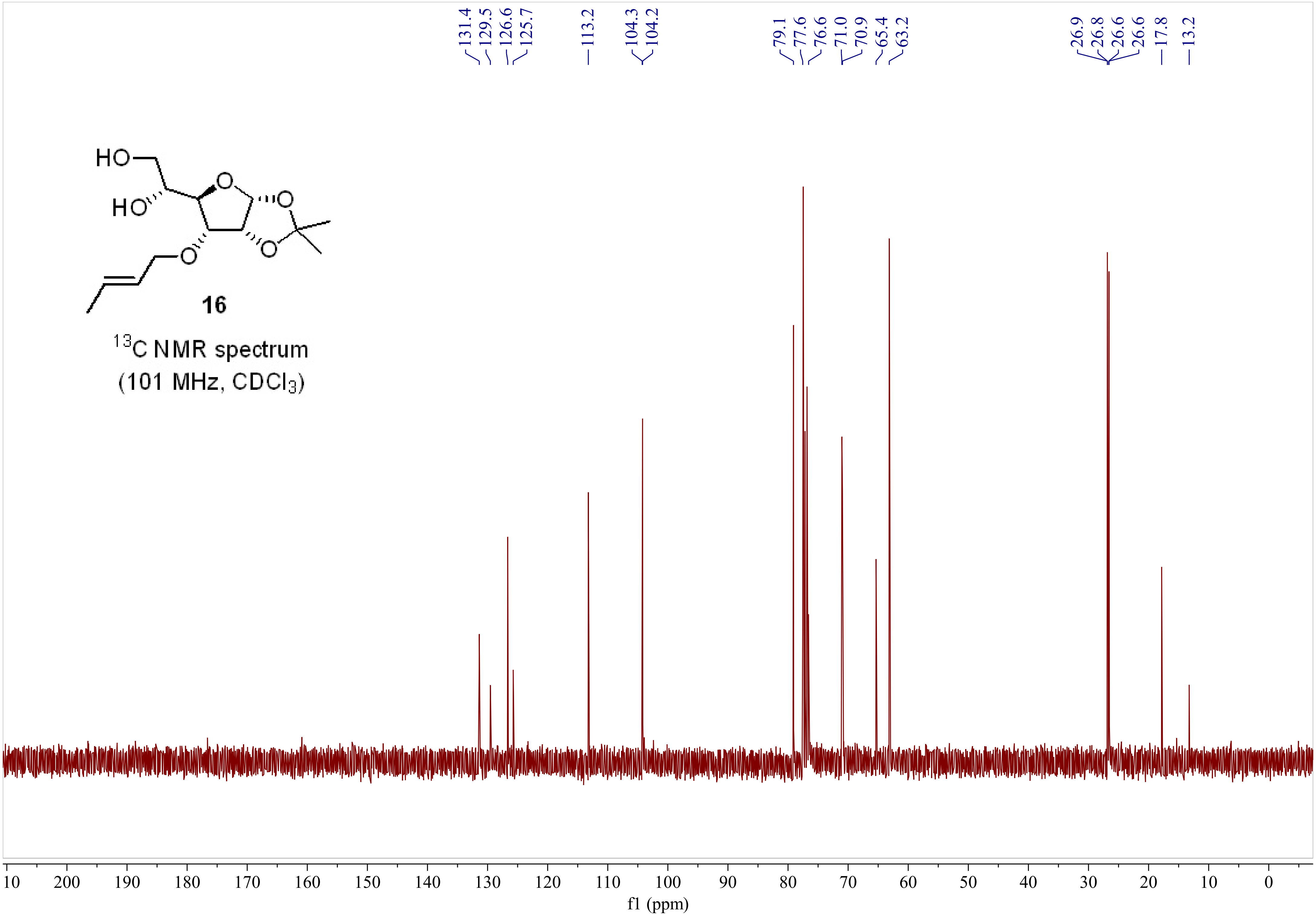
131.4
129.5
126.6
125.7
113.2
104.3
104.2
79.1
77.6
76.6
71.0
70.9
65.4
63.2
26.9
26.8
26.6
26.6
17.8
13.2
HO
HO
O
O
O
O
16
^{13}C NMR spectrum
(101 MHz, $CDCl_3$)
10
200
190
180
170
160
150
140
130
120
110
100
90
80
70
60
50
40
30
20
10
0
f1 (ppm)

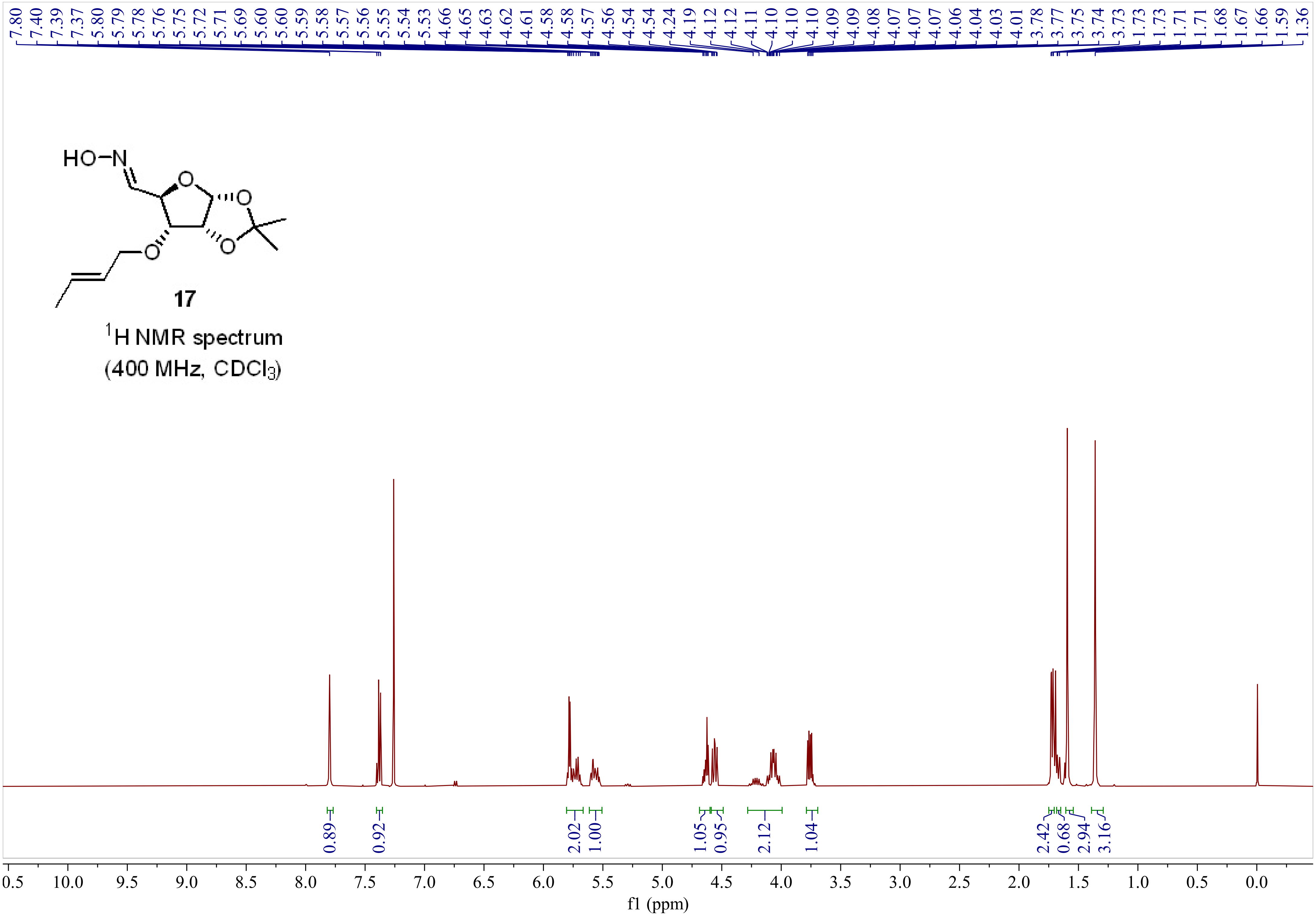

HO−N
17
^{1}H NMR spectrum
(400 MHz, $CDCl_3$)
7.80 7.40 7.39 7.37 5.80 5.79 5.78 5.76 5.75 5.72 5.71 5.69 5.60 5.60 5.59 5.58 5.57 5.56 5.55 5.54 5.53 4.66 4.65 4.63 4.62 4.61 4.58 4.58 4.57 4.56 4.54 4.54 4.24 4.19 4.12 4.12 4.11 4.10 4.10 4.10 4.09 4.09 4.08 4.07 4.07 4.07 4.06 4.04 4.03 4.01 3.78 3.77 3.75 3.74 3.73 1.73 1.73 1.71 1.71 1.68 1.67 1.66 1.59 1.36
0.89 0.92 2.02 1.00 1.05 0.95 2.12 1.04 2.42 0.68 2.94 3.16
0.5 10.0 9.5 9.0 8.5 8.0 7.5 7.0 6.5 6.0 5.5 5.0 4.5 4.0 3.5 3.0 2.5 2.0 1.5 1.0 0.5 0.0
f1 (ppm)

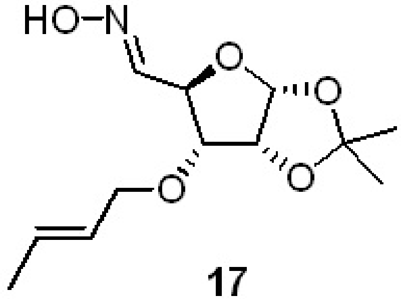

17

^{13}C NMR spectrum
(101 MHz, $CDCl_3$)

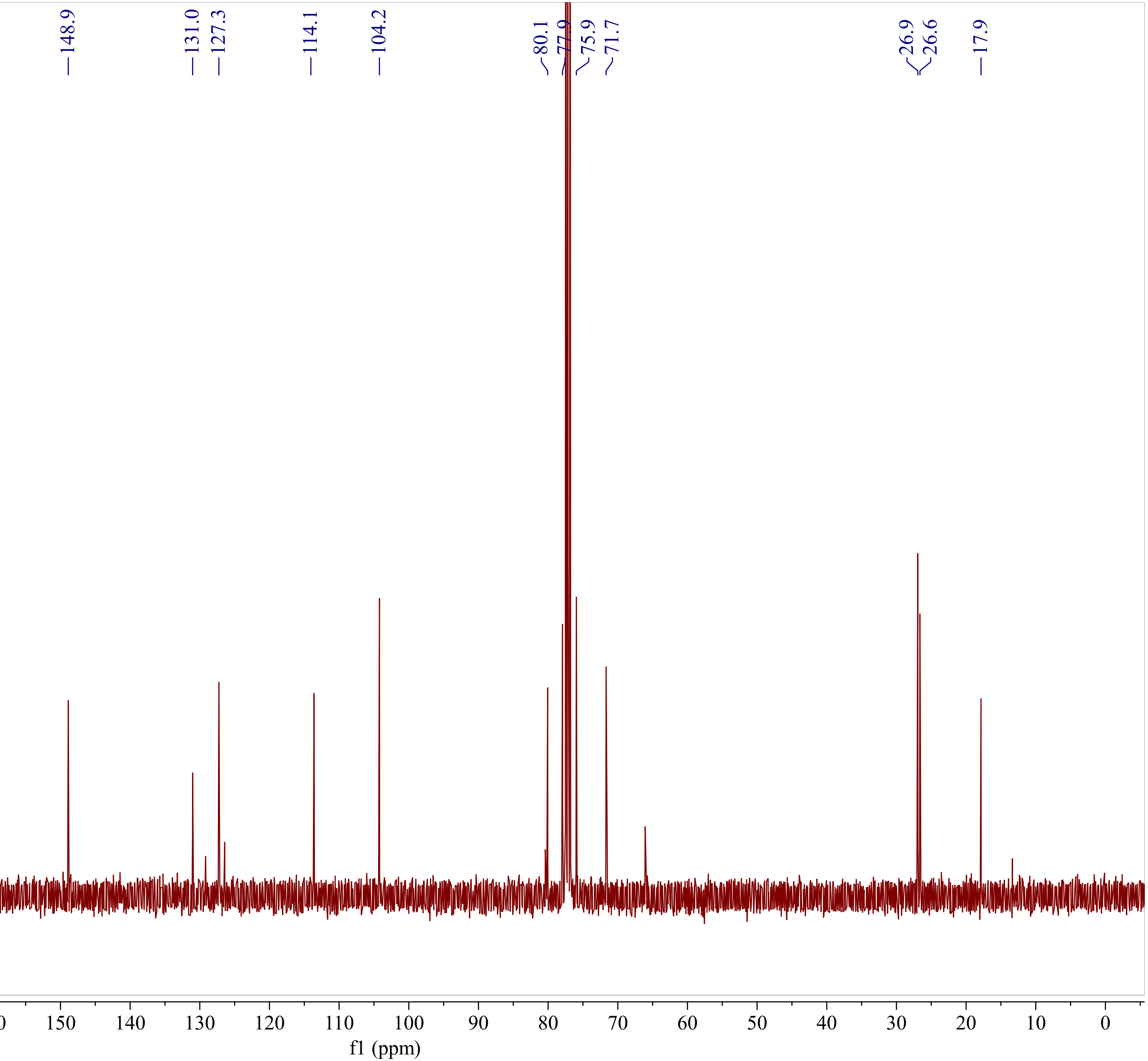

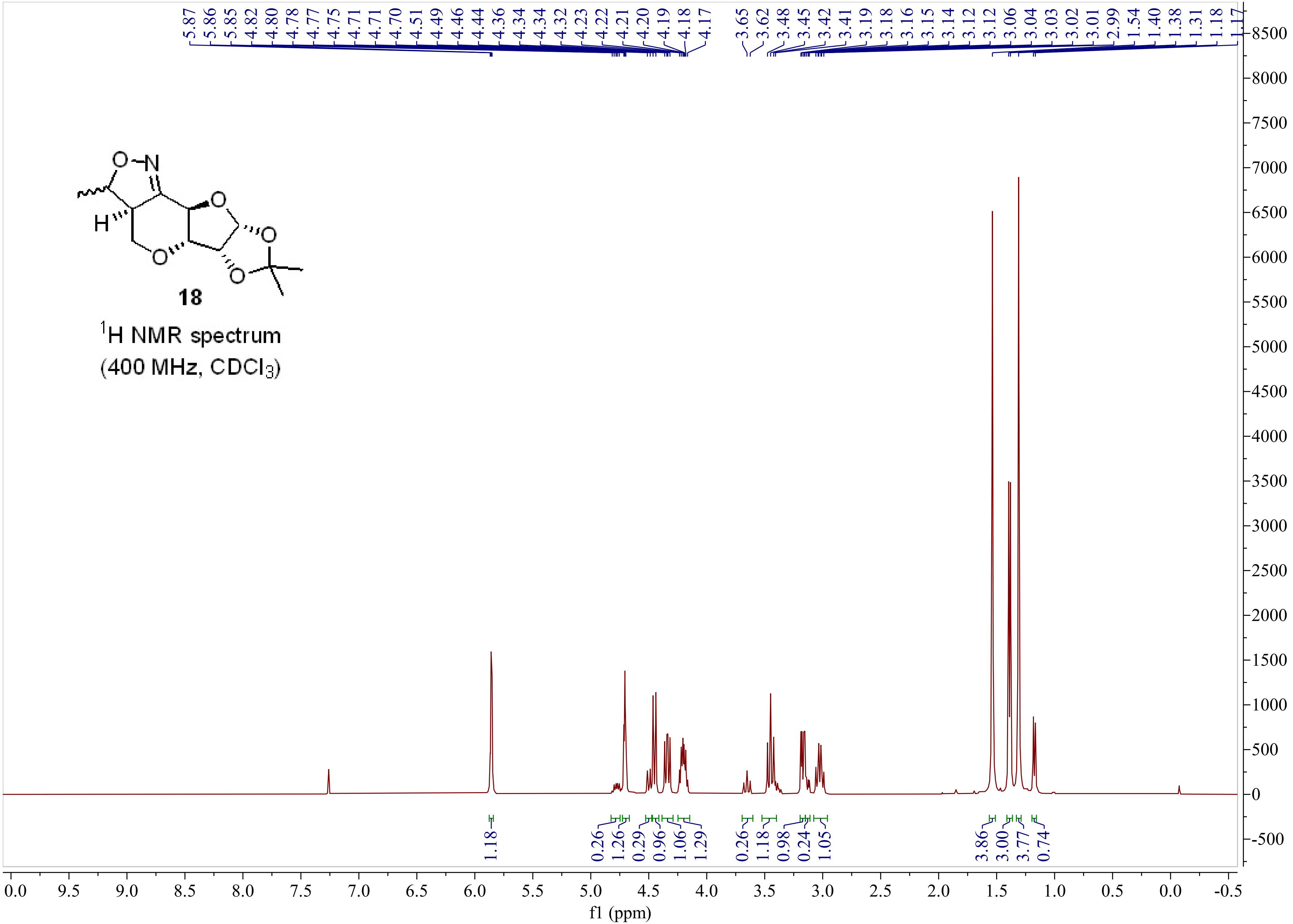
5.87 5.86 5.85 4.82 4.80 4.78 4.77 4.75 4.71 4.71 4.70 4.51 4.49 4.46 4.44 4.36 4.34 4.34 4.32 4.23 4.22 4.21 4.20 4.19 4.18 4.17
3.65 3.62 3.48 3.45 3.42 3.41 3.19 3.18 3.16 3.15 3.14 3.12 3.12 3.06 3.04 3.03 3.02 3.01 2.99 1.54 1.40 1.38 1.31 1.18 1.17
O–N
H
O
18
1H NMR spectrum
(400 MHz, CDCl3)
1.18
0.26 1.26 0.29 0.96 1.06 1.29
0.26 1.18 0.98 0.24 1.05
3.86 3.00 3.77 0.74
f1 (ppm)

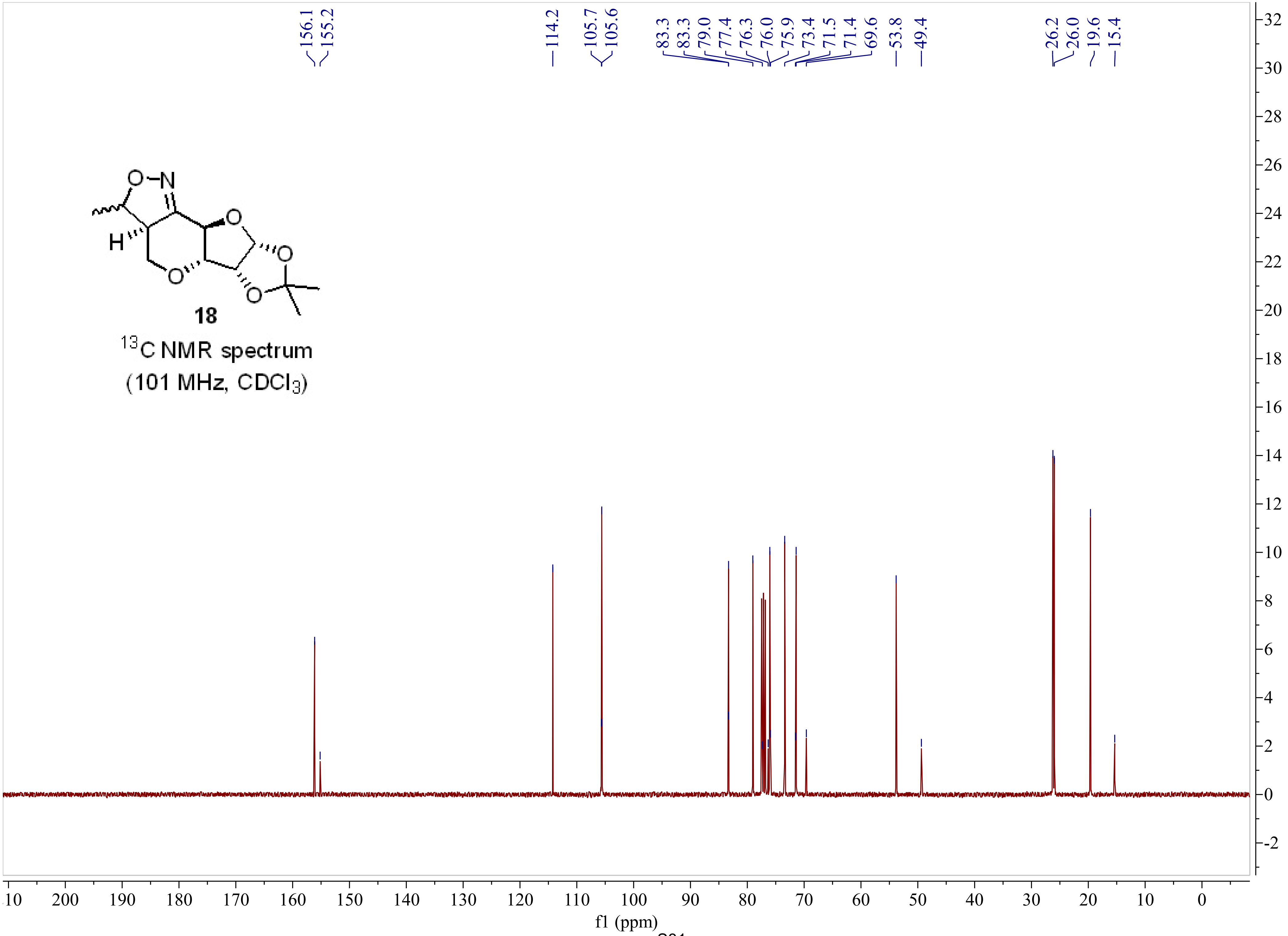
156.1
155.2
114.2
105.7
105.6
83.3
83.3
79.0
77.4
76.3
76.0
75.9
73.4
71.5
71.4
69.6
53.8
49.4
26.2
26.0
19.6
15.4
O
N
H
O
O
O
O
18
^{13}C NMR spectrum
(101 MHz, $CDCl_3$)
f1 (ppm)

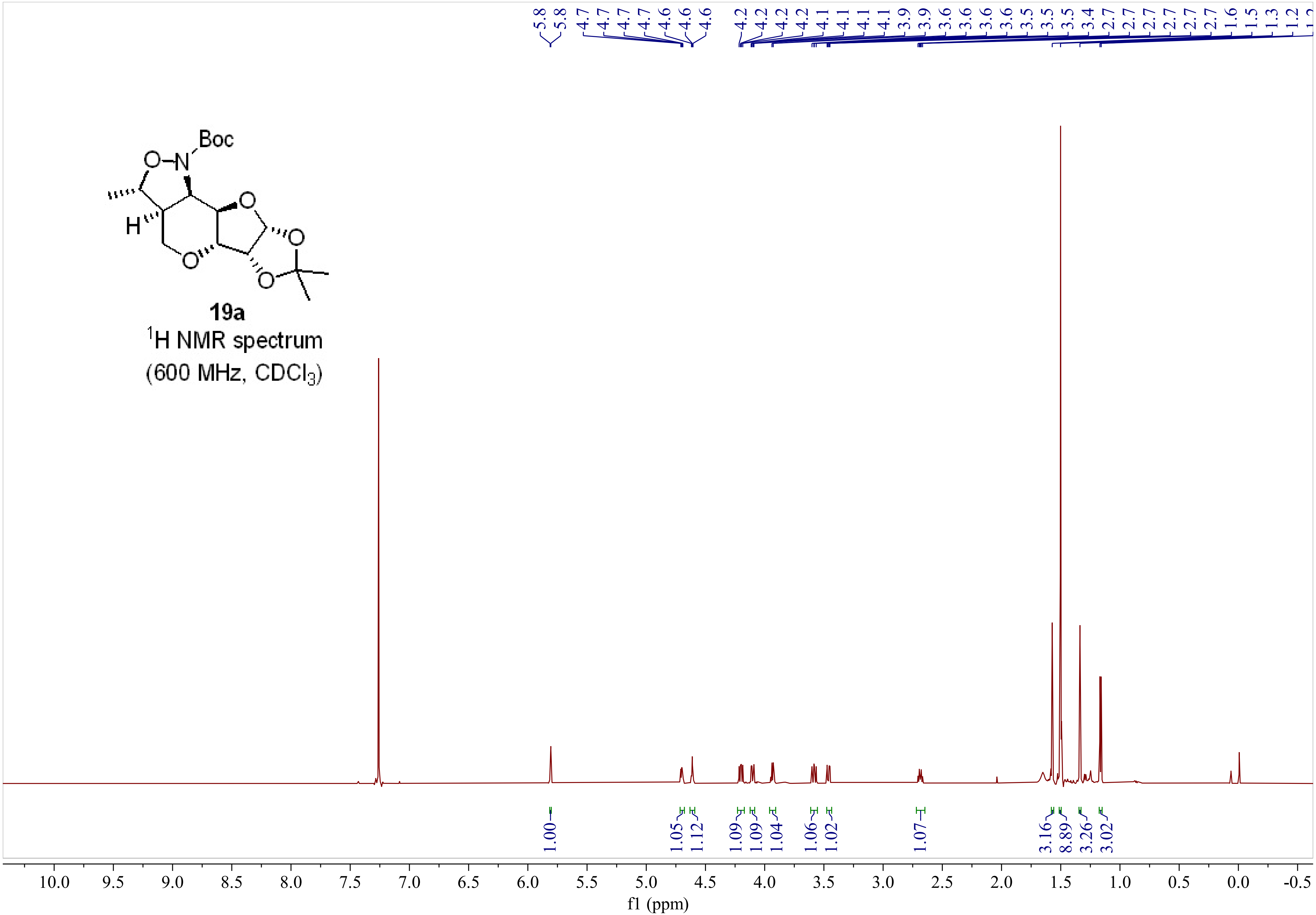
Boc
H
19a
^{1}H NMR spectrum
(600 MHz, $CDCl_3$)
1.00
1.05
1.12
1.09
1.09
1.04
1.06
1.02
1.07
3.16
8.89
3.26
3.02
10.0 9.5 9.0 8.5 8.0 7.5 7.0 6.5 6.0 5.5 5.0 4.5 4.0 3.5 3.0 2.5 2.0 1.5 1.0 0.5 0.0 -0.5
f1 (ppm)

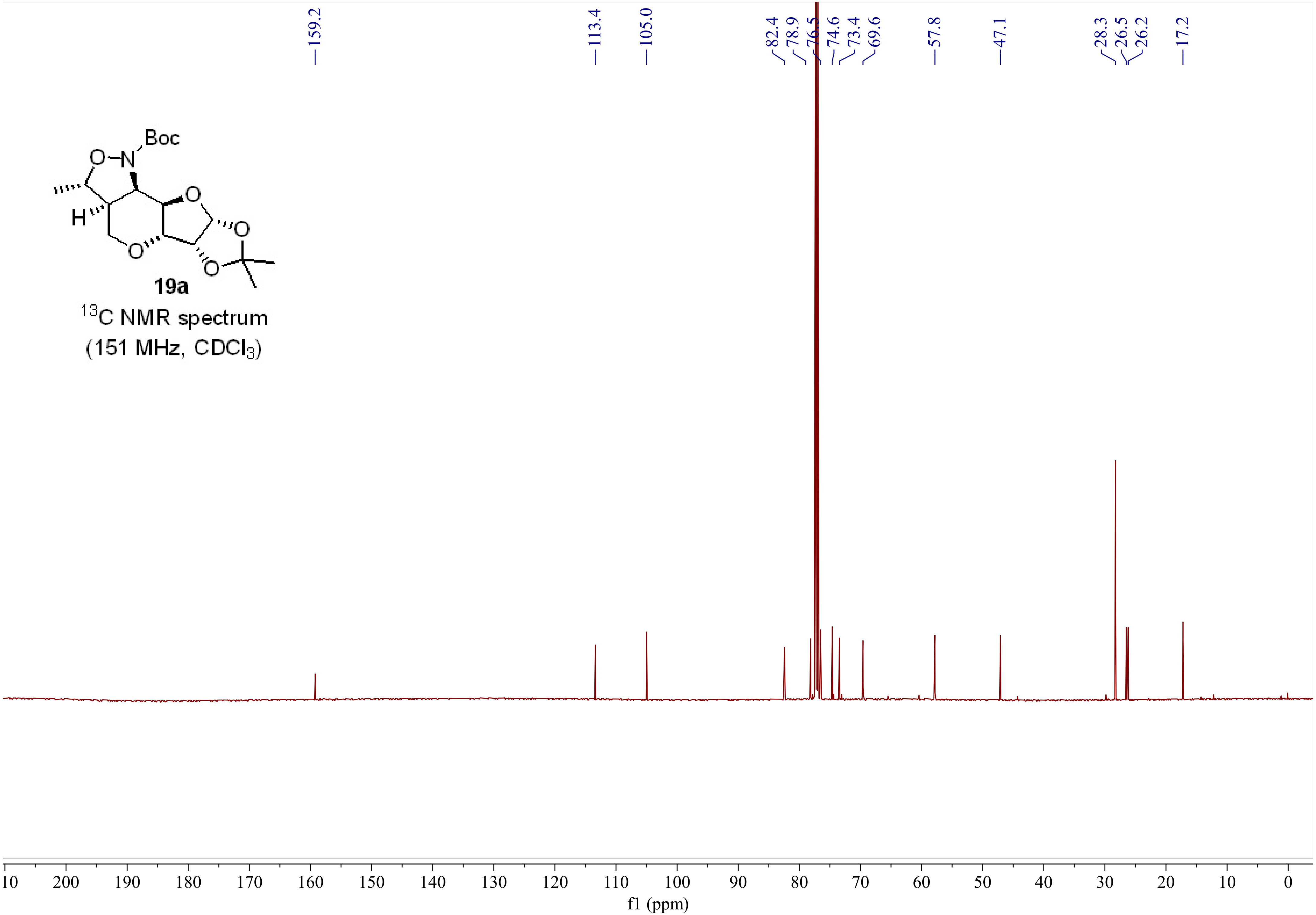
Boc
O
N
O
H
O
O
O
19a
^{13}C NMR spectrum
(151 MHz, $CDCl_3$)
159.2
113.4
105.0
82.4
78.9
76.5
74.6
73.4
69.6
57.8
47.1
28.3
26.5
26.2
17.2
10 200 190 180 170 160 150 140 130 120 110 100 90 80 70 60 50 40 30 20 10 0
f1 (ppm)

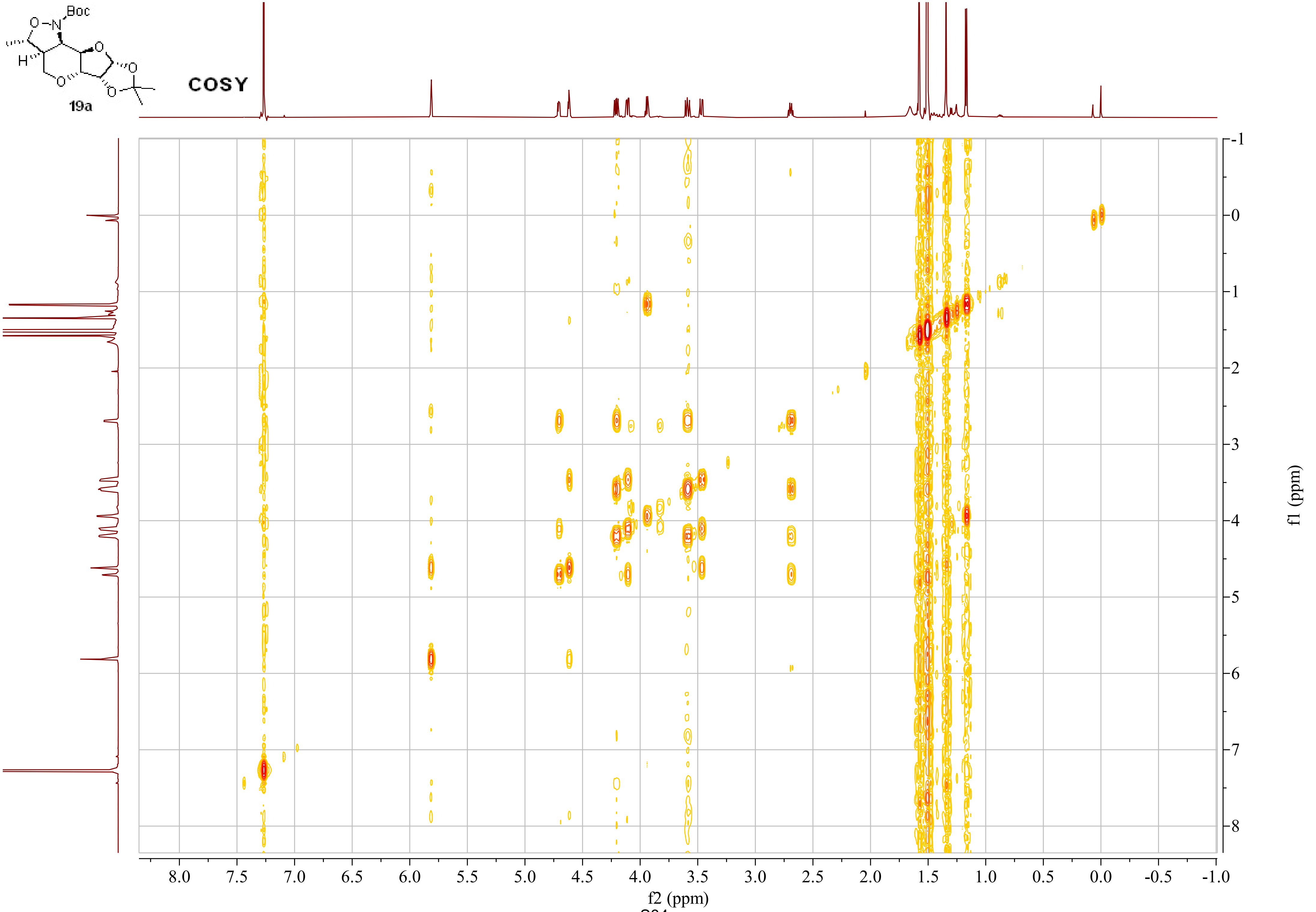
Boc
19a
COSY
f2 (ppm)
f1 (ppm)

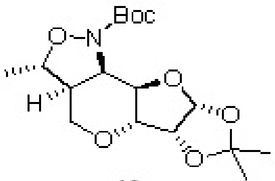

19a

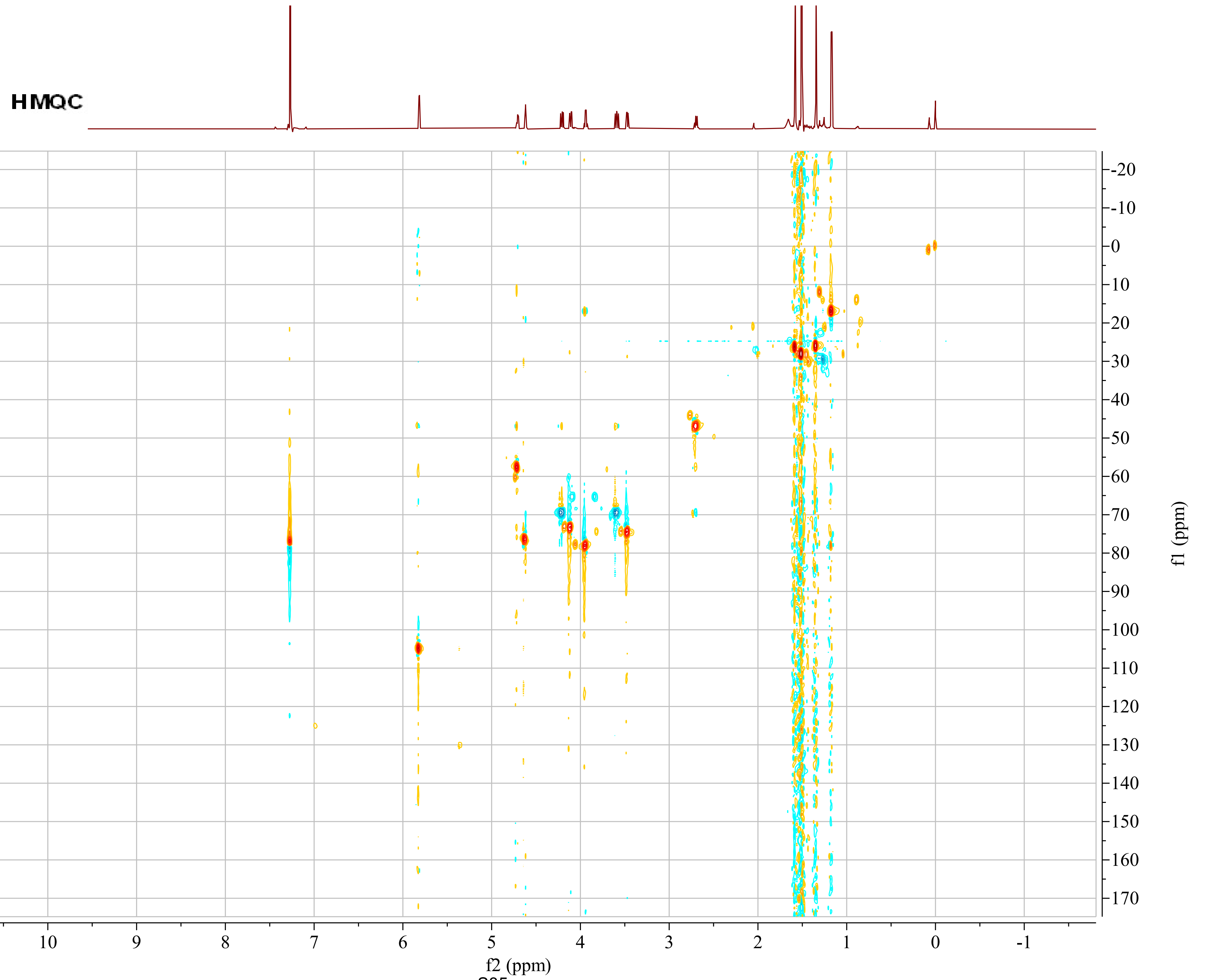

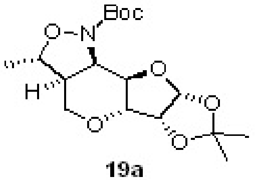

19a

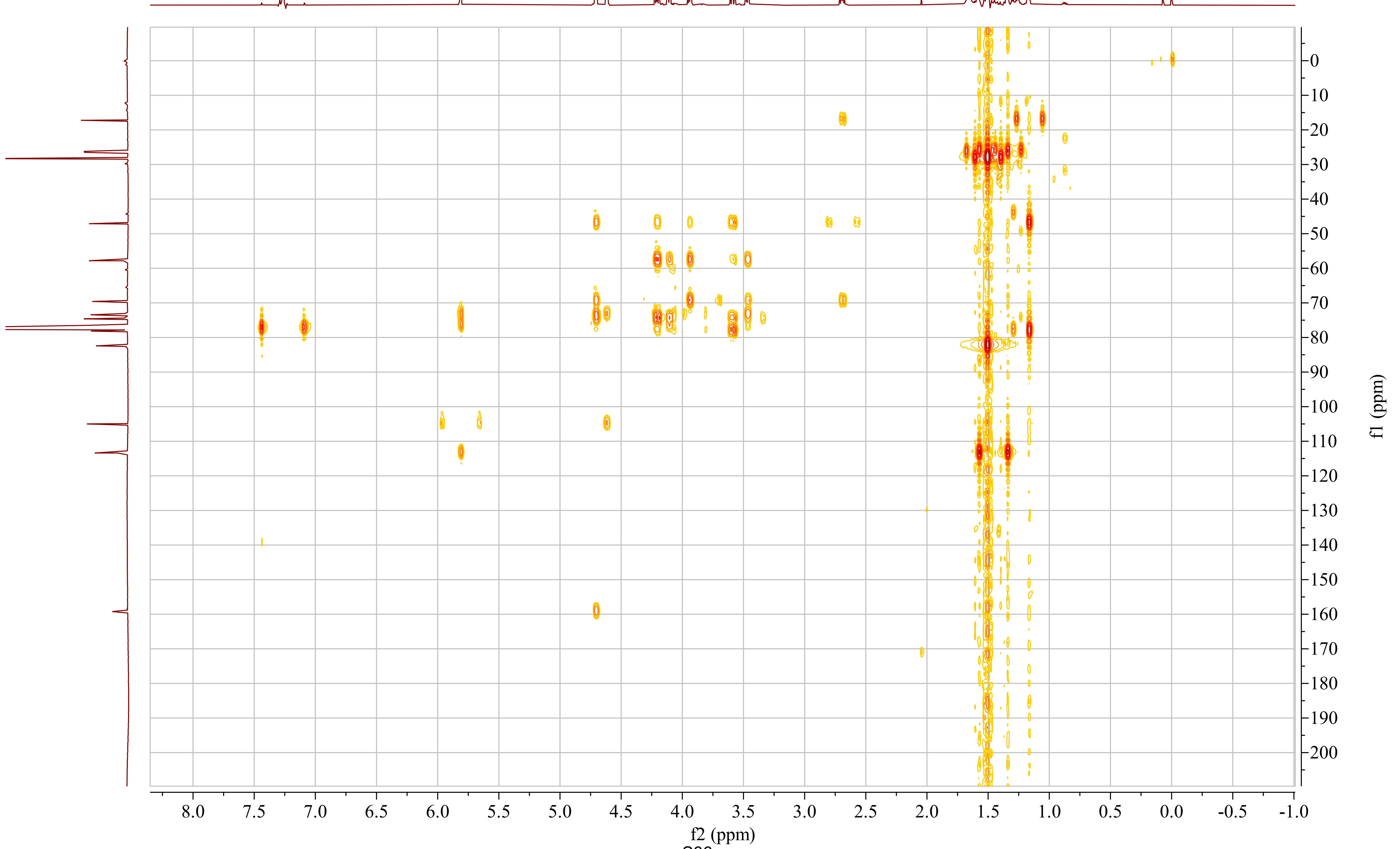

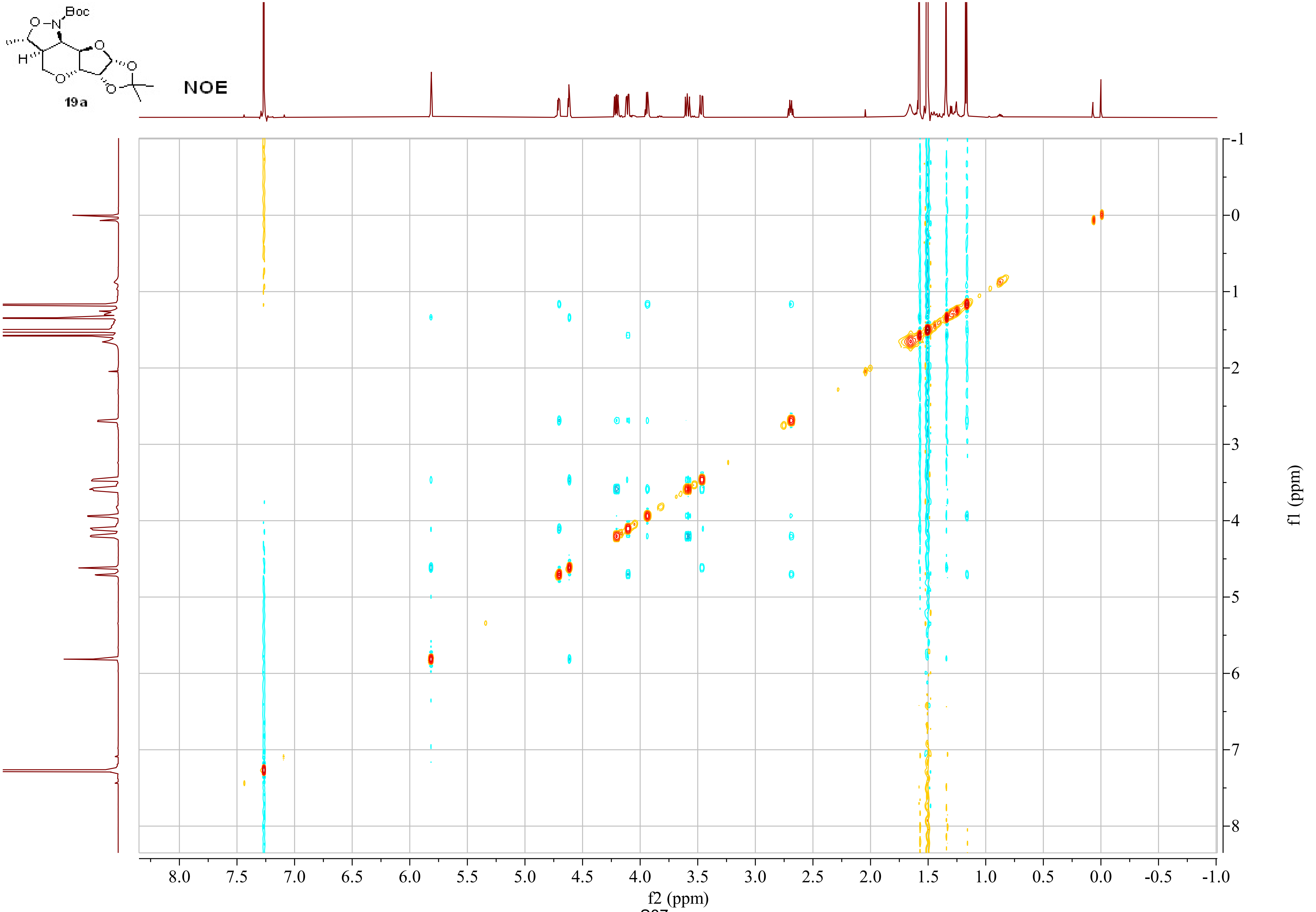

Boc
O–N
H
O
O
O
O
19a
NOE
f2 (ppm)
f1 (ppm)

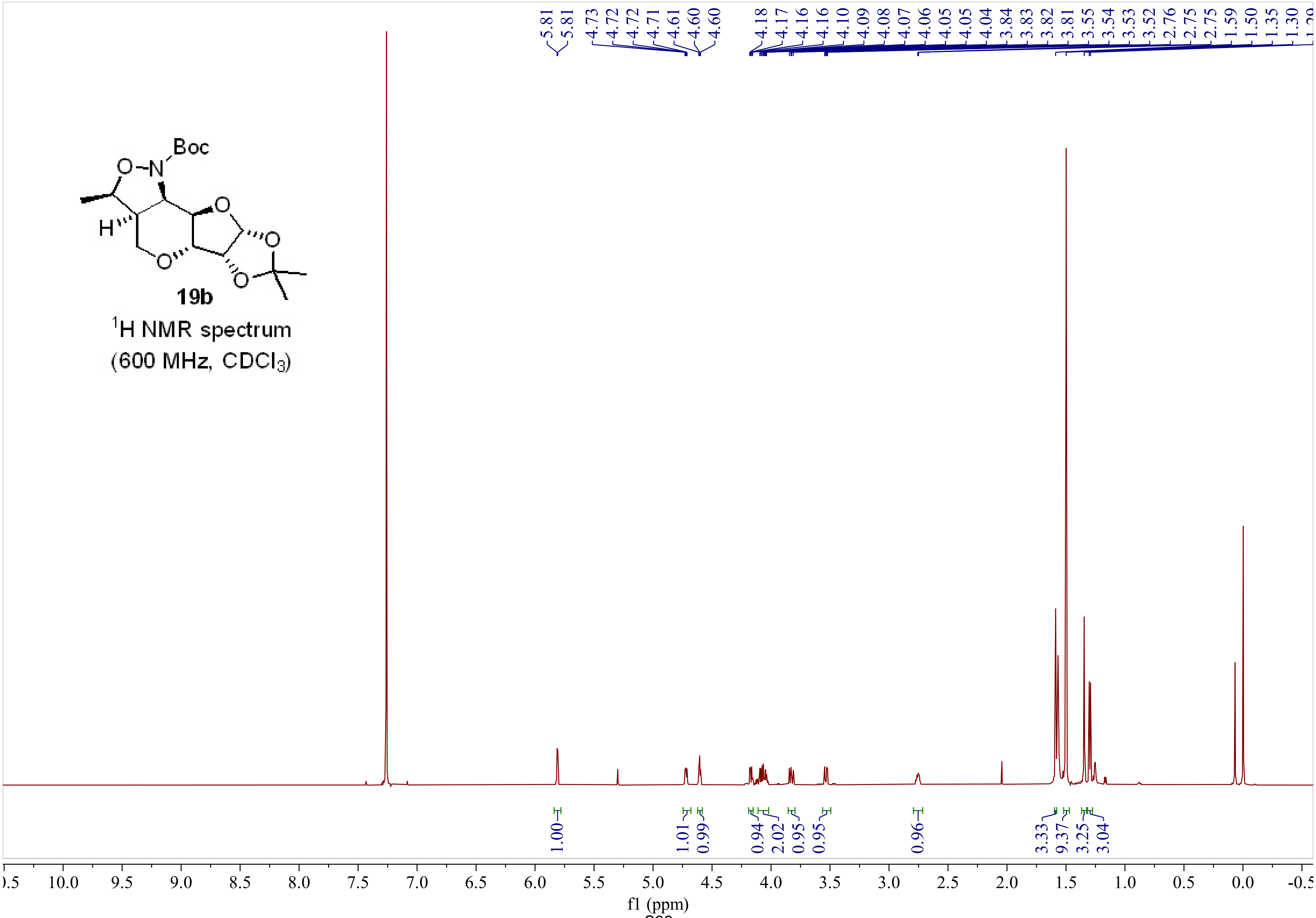
5.81
5.81
4.73
4.72
4.72
4.71
4.61
4.60
4.60
4.18
4.17
4.16
4.16
4.10
4.09
4.08
4.07
4.06
4.05
4.05
4.04
3.84
3.83
3.82
3.81
3.55
3.54
3.53
3.52
2.76
2.75
2.75
1.59
1.50
1.35
1.30
Boc
O
N
H
O
O
O
O
19b
^{1}H NMR spectrum
(600 MHz, $CDCl_3$)
1.00
1.01
0.99
0.94
2.02
0.95
0.95
0.96
3.33
9.37
3.25
3.04
10.0
9.5
9.0
8.5
8.0
7.5
7.0
6.5
6.0
5.5
5.0
4.5
4.0
3.5
3.0
2.5
2.0
1.5
1.0
0.5
0.0
f1 (ppm)

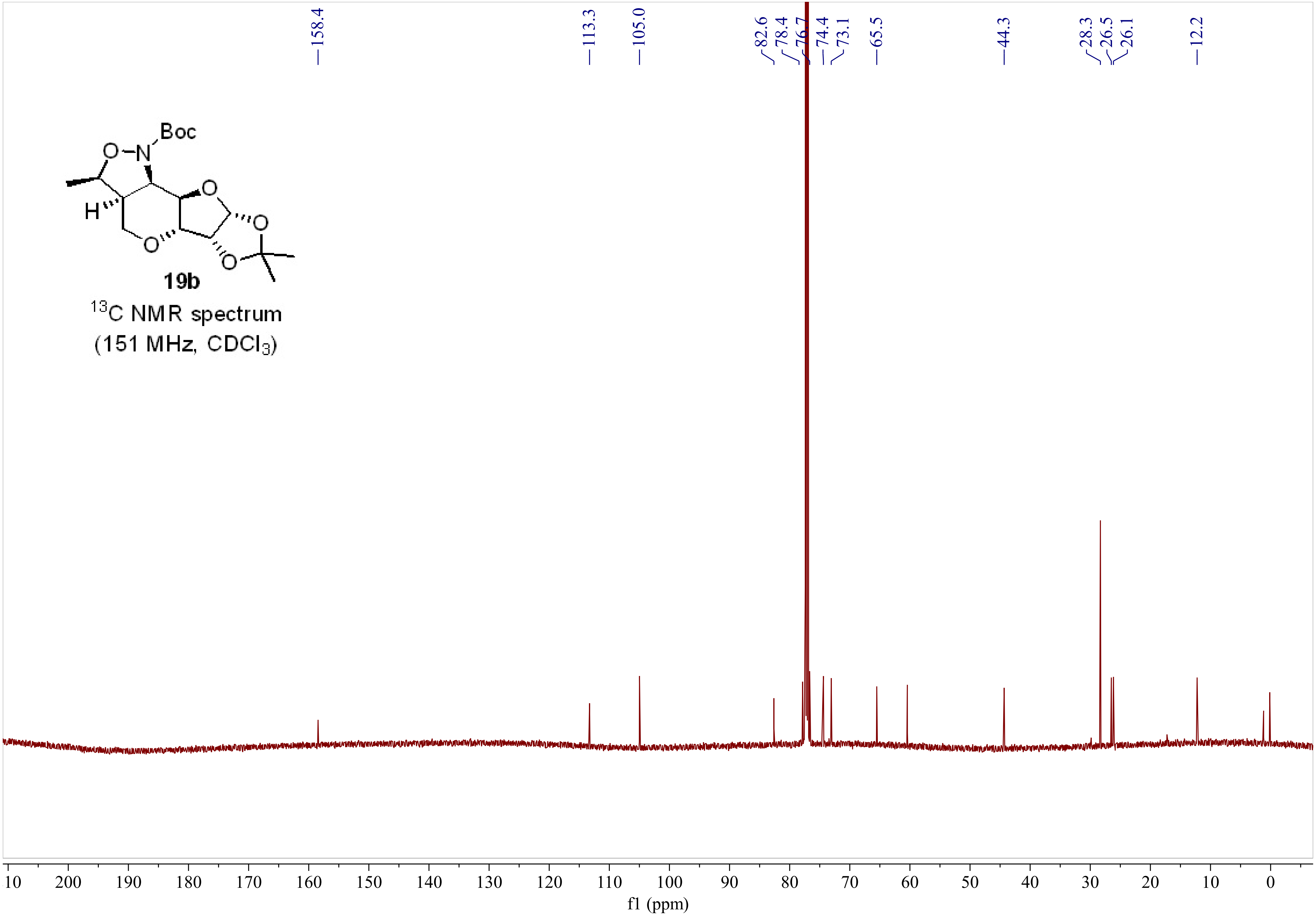

158.4
113.3
105.0
82.6
78.4
76.7
74.4
73.1
65.5
44.3
28.3
26.5
26.1
12.2
Boc
O
N
H
19b
^{13}C NMR spectrum
(151 MHz, $CDCl_3$)
210
200
190
180
170
160
150
140
130
120
110
100
90
80
70
60
50
40
30
20
10
0
f1 (ppm)

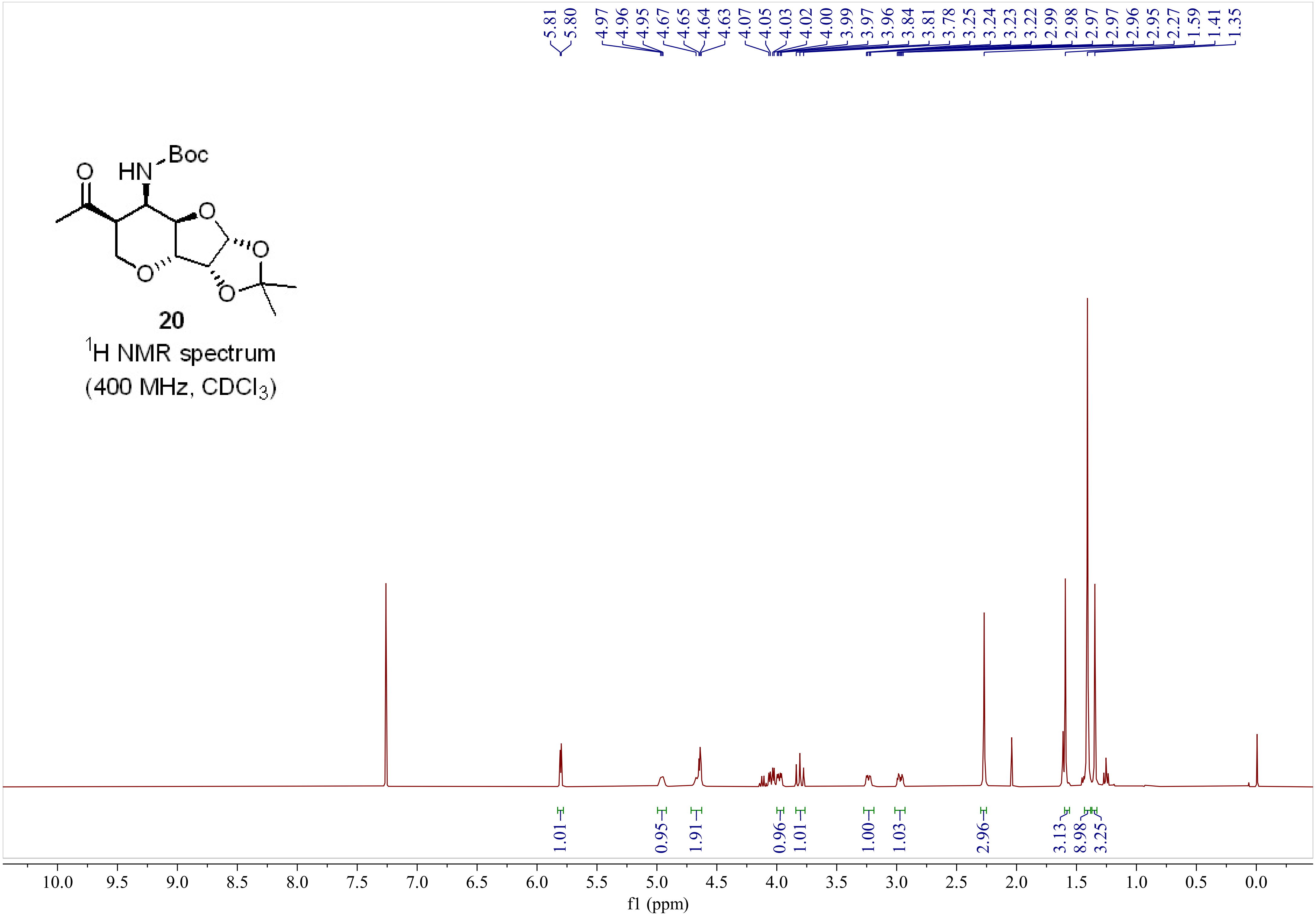
5.81
5.80
4.97
4.96
4.95
4.67
4.65
4.64
4.63
4.07
4.05
4.03
4.02
4.00
3.99
3.97
3.96
3.84
3.81
3.78
3.25
3.24
3.23
3.22
2.99
2.98
2.97
2.97
2.96
2.95
2.27
1.59
1.41
1.35
HN
Boc
O
O
O
O
O
20
1H NMR spectrum
(400 MHz, CDCl3)
1.01
0.95
1.91
0.96
1.01
1.00
1.03
2.96
3.13
8.98
3.25
10.0 9.5 9.0 8.5 8.0 7.5 7.0 6.5 6.0 5.5 5.0 4.5 4.0 3.5 3.0 2.5 2.0 1.5 1.0 0.5 0.0
f1 (ppm)

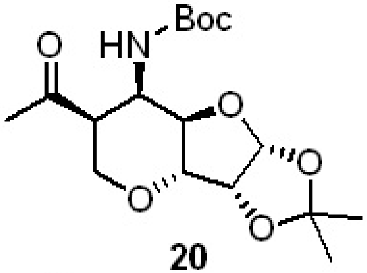

20

^{13}C NMR spectrum

(101 MHz, $CDCl_3$)

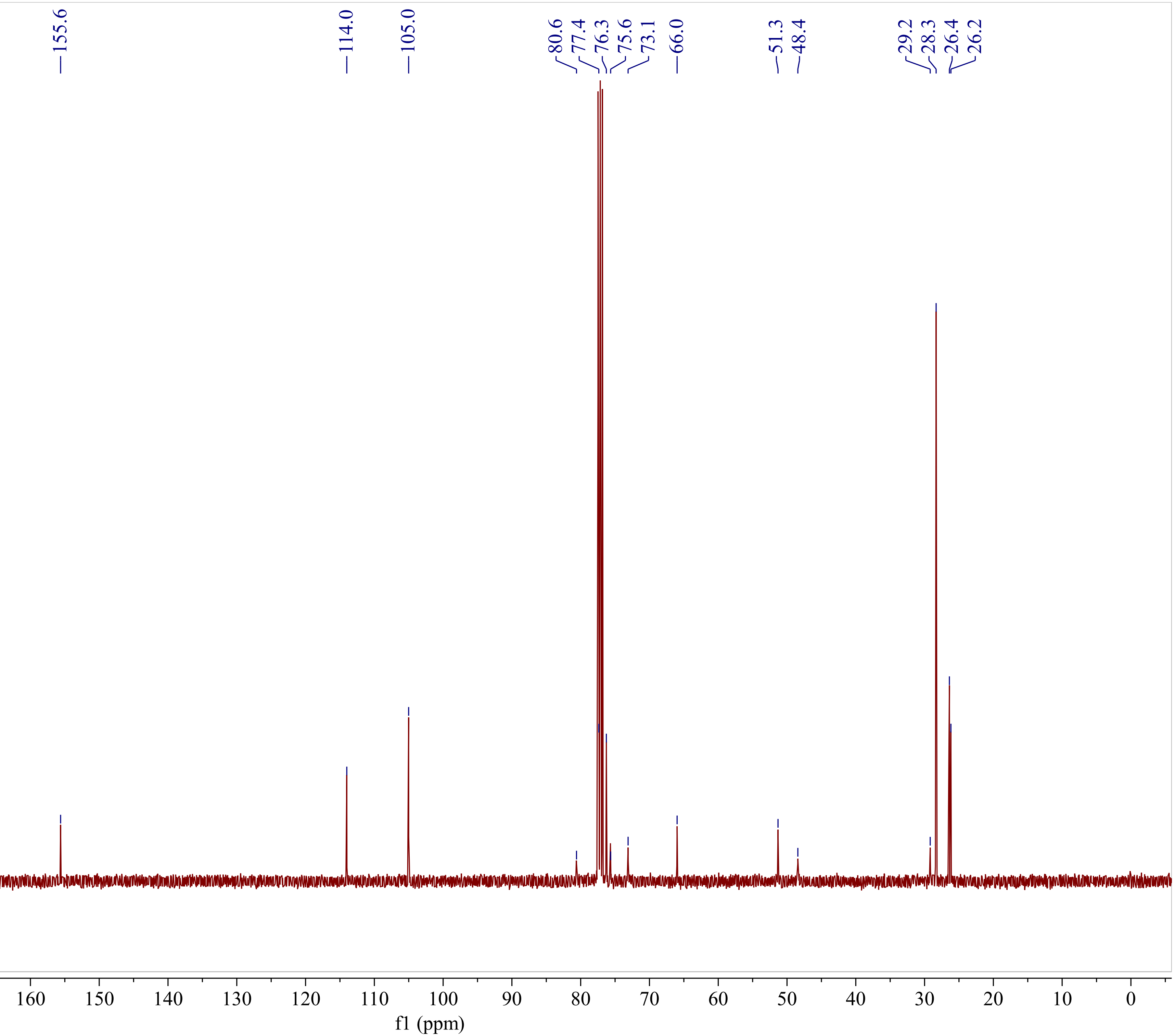

Supplement: File 1 — Experimental procedures and compound characterization data. [file Beilstein_J_Org_Chem-21-2542-s001.pdf]
